# TMT-Based Proteomic Analysis of Plasma from Children with Rolandic Epilepsy

**DOI:** 10.1155/2020/8840482

**Published:** 2020-10-07

**Authors:** Ji Sun, Tiechao Jiang, Feng Gu, Dihui Ma, Jianmin Liang

**Affiliations:** ^1^Department of Pediatric Neurology, The First Hospital of Jilin University, Changchun, Jilin 130021, China; ^2^Department of Cardiology, The China-Japan Union Hospital of Jilin University, Changchun, Jilin 130031, China; ^3^Department of Infectious Diseases, The People's Hospital of Jilin Province, Changchun, Jilin 130021, China; ^4^Department of Neurology, The First Hospital of Jilin University, Changchun, Jilin 130021, China

## Abstract

Rolandic epilepsy is one of the most common epileptic syndromes in childhood. We used TMT-based proteomics and bioinformatics analysis to identify the differentially expressed proteins in plasma of children with Rolandic epilepsy. Our aim was to provide a molecular basis for exploring possible mechanisms underlying the pathogenesis of epilepsy. Subjects were divided into two groups (five in each): patients with Rolandic epilepsy as cases and patients with migraine as controls. Total proteins were extracted and quantitatively labeled with TMT, then analyzed using liquid chromatography mass spectrometry. Bioinformatics analysis was used to identify the hub genes. A total of 752 proteins were identified, of which 670 contained quantitative proteins. 217 differentially expressed proteins were identified, 46 of which were only upregulated in more than two groups and 111 of which were only downregulated in more than two groups. Bioinformatics analysis revealed top 10 hub genes in the up- and downregulated groups, respectively. Our study demonstrates that some differentially expressed proteins are associated with epilepsy. Activation of acute-phase or innate immune response and complement and fibrinogen systems and repression of glycolysis, lipoprotein metabolism, and antioxidant activity may play a role in the development of epilepsy.

## 1. Introduction

Rolandic epilepsy is the most common focal-onset epilepsy in childhood. The age at onset ranges from 3 to 13 years. It typically includes partial orofacial motor or sensory seizures, with or without secondary generalization, occurring most often during sleep or on awakening. Most cases of Rolandic epilepsy are idiopathic, but sometimes, they can be of symptomatic origin [1].

The diagnosis of epilepsy is mainly based on reliable clinical and historical features and EEG. However, the clinical and historical features sometimes are atypical, which make diagnosis difficult. Therefore, effective diagnostic tools and identification of disease biomarkers would be approved to facilitate correct clinical diagnosis as well as to generate treatments to prevent or cure epilepsy.

Plasma is the primary clinical specimen and represents the largest version of the human proteome present in any sample. It contains not only the classical “plasma proteins” but also all tissue proteins (as leakage markers) and numerous distinct immunoglobulin sequences. The human plasma proteome using multidimensional survey techniques has provided major challenges in proteomics during the past few years [2].

Nowadays, there have been few reports on the use of plasma proteome for seizure biomarker identification in children with Rolandic epilepsy. This was the reason why we performed this trial to discover a panel of disease-specific biomarkers of Rolandic epilepsy from patients' plasma that seemed to contribute to illustrate the mechanisms of epileptogenesis and to be a useful tool for epilepsy diagnosis.

## 2. Materials and Methods

### 2.1. Subjects

All the individuals including the epilepsy group and the control group were recruited from May 1, 2017, to Dec 1, 2018, at random. We labeled the patients with Rolandic epilepsy as E1 to E5 and the patients with migraine as C1 to C5. The epilepsy group consisted of five patients ranging from7 to 11 years old (mean ± SD, 9.28 ± 1.30 years) with three men and two women. All patients underwent a comprehensive clinical examination, including a medical history, neurological examination, routine laboratory tests, EEG, and brain MRI. Epilepsy was diagnosed as Rolandic epilepsy according to the criteria proposed by the International League Against Epilepsy (ILAE) in 2001 [3] and had no underlying etiology. All patients had normal brain MRI and never taken any antiepilepsy drugs (AEDs).

The control group consisted of five sex- and age-matched individuals who were diagnosed as having migraine according to the criteria proposed by the International Headache Society (IHS) in 2018 [4]. The demographic and disease-related characteristics of the patients and controls are shown in Table 1. All people recruited in the research were of Chinese origin. Our hospital's Research Ethics Board approved this study (project identification code: 2017-295). Written informed consent was obtained from parents or guardians before recruitment.

## 3. Protein Extraction and Identification

### 3.1. Plasma Samples

For each subject, 5 ml of venous blood samples was collected. Firstly, the blood samples were centrifuged at 2000 × g for 15 min at 4°C to collect the plasma, which was then subpackaged and stored at -80°C until further analysis. Secondly, the cellular debris of the plasma sample was removed by centrifugation at 12000 × g for 10 min at 4°C. Then, the supernatant was transferred to a new centrifuge tube. The top 12 high-abundance proteins were removed by Pierce™ Top 12 Abundant Protein Depletion Spin Columns Kit (ThermoFisher Scientific, Waltham, MA, USA). Finally, the protein concentration was determined with a BCA kit (Beyotime, Jiangsu, China) according to the manufacturer's instructions.

### 3.2. Trypsin Digestion

For digestion, the protein solution was reduced with 5 mM dithiothreitol for 30 min at 56°C and alkylated with 11 mM iodoacetamide for 15 min at room temperature in darkness. The protein sample was then diluted by adding 100 mM TEAB to urea concentration less than 2 M. Finally, trypsin was added at 1 : 50 trypsin-to-protein mass ratio for the first digestion overnight and 1 : 100 trypsin-to-protein mass ratio for a second 4 h digestion.

### 3.3. TMT Labeling

After trypsin digestion, peptide was desalted by Strata-X C18 SPE column (Phenomenex, Torrance, CA, USA) and vacuum-dried. Peptide was reconstituted in 0.5 M TEAB and processed according to the manufacturer's protocol for a TMT (Tandem Mass Tag) kit (ThermoFisher Scientific, Waltham, MA, USA). Briefly, one unit of TMT reagent was thawed and reconstituted in acetonitrile. The peptide mixtures were then incubated for 2 h at room temperature and pooled, desalted, and dried by vacuum centrifugation.

### 3.4. HPLC Fractionation

The tryptic peptides were fractionated into fractions by high-pH reverse-phase HPLC (high-performance liquid chromatography) using Agilent 300Extend C18 column (5 *μ*m particles, 4.6 mm ID, 250 mm length, Agilent, Santa Clara, CA, USA). Briefly, peptides were first separated with a gradient of 8% to 32% acetonitrile (pH 9.0) over 60 min into 60 fractions. Then, the peptides were combined into 18 fractions and dried by vacuum centrifuging.

### 3.5. LC-MS/MS Analysis

The tryptic peptides were dissolved in 0.1% formic acid (solvent A), directly loaded onto a homemade reversed-phase analytical column (15 cm length, 75 *μ*m ID). The gradient was comprised of an increase from 9% to 24% solvent B (0.1% formic acid in 90% acetonitrile) over 38 min, 24% to 35% in 14 min, and climbing to 80% in 4 min then holding at 80% for the last 4 min, all at a constant flow rate of 700 nl/min on an EASY-nLC 1000 UPLC (ultraperformance liquid chromatography) system (ThermoFisher Scientific, Waltham, MA, USA). The peptides were subjected to NSI source followed by tandem mass spectrometry (MS/MS) in Q Exactive™ Plus (ThermoFisher Scientific, Waltham, MA, USA) coupled online to the UPLC. The electrospray voltage applied was 2.0 kV. The *m*/*z* scan range was 350 to 1800 for full scan, and intact peptides were detected in the Orbitrap at a resolution of 70,000. Peptides were then selected for MS/MS using NCE setting as 28, and the fragments were detected in the Orbitrap at a resolution of 17,500. A data-dependent procedure alternated between one MS scan and 20 MS/MS scans with 15.0 s dynamic exclusion. Automatic gain control (AGC) was set at 5E4. Fixed first mass was set as 100 *m*/*z*.

### 3.6. Database Searching

The resulting MS/MS data were processed using the MaxQuant search engine (v.1.5.2.8). Tandem mass spectra were searched against the Swiss-Prot human database concatenated with a reverse decoy database. Trypsin/P was specified as a cleavage enzyme allowing up to 2 missing cleavages. The mass tolerance for precursor ions was set as 20 ppm in the first search and 5 ppm in the main search, and the mass tolerance for fragment ions was set as 0.02 Da. FDR was adjusted to 1%.

## 4. Bioinformatics Analysis

Differentially expressed proteins with expression log2 fold change > 1.2 and *p* value < 0.05 were selected as candidate proteins for further analysis. Then, Gene Ontology (GO) annotation proteome was derived from the UniProt-GOA database (http://www.ebi.ac.uk/GOA/). If some identified proteins were not annotated by the UniProt-GOA database, the InterProScan software would be used to annotate the protein's GO function based on a protein sequence alignment method. Proteins were classified by GO annotation into three categories: biological process, cellular compartment, and molecular function. For each category, a two-tailed Fisher exact test was employed to test the enrichment of the differentially expressed protein against all identified proteins. The GO with a corrected *p* value < 0.05 is considered significant. The KEGG (Kyoto Encyclopedia of Genes and Genomes, https://http://www.kegg.jp/) database was used to identify enriched KEGG pathways by a two-tailed Fisher exact test to test the enrichment of the differentially expressed protein against all identified proteins [5]. The results were filtered by the following criteria: a corrected *p* value < 0.05 and protein counts > 5. The protein–protein interaction (PPI) networks of the differentially expressed proteins were established based on STRING (Search Tool for the Retrieval of Interacting Genes, https://string-db.org) [6]. We set confidence score ≥ 0.4 and the maximum number of interactors = 0 as the cutoff criterion. Then, the top 10 hub genes of the differentially expressed proteins were screened by the cytoHubba from the Cytoscape 3.7.0 (http://www.cytoscape.org/) platform according to the high degree of connectivity [7].

## 5. Statistical Analysis

All experimental data were presented as means ± SD using SPSS software (version 13.0, SPSS).

## 6. Results

### 6.1. TMT-Based Quantitative Proteomic Basis Data Analysis and Overall Protein Identification

When plasma of patients with Rolandic epilepsy was compared with that of controls using TMT-based quantitative proteomics, a total of 213,300 (17,617 matched) spectra were obtained. Among these spectra, 4659 identified peptides (4541 unique peptides) and 752 identified proteins (670 quantified proteins) were detected (Table S1), and the average peptides mass error was <10 ppm, indicating a high mass accuracy of the MS data (Figure S1a). The lengths of most identified peptides were 8 to 20 amino acid residues (Figure S1b), suggesting that our sampling met the required standard. The detailed information of identified proteins, including protein accession, protein description, gene name, protein molecular weight, peptide number, and matching scores, is shown in Table S2.

### 6.2. Identification of Differentially Expressed Proteins

Differentially expressed proteins (DEPs) were defined as those with a >1.2-fold change in relative abundance (*p* < 0.05) between five patients and controls, respectively. In total, 217 differentially expressed proteins were identified, 46 of which were only upregulated in more than two groups (patient vs. control) and 111 of which were only downregulated in more than two groups. The detailed information of differentially expressed proteins is shown in Table S3.

### 6.3. Enrichment Analysis of DEPs in GO

**Figure 1:**
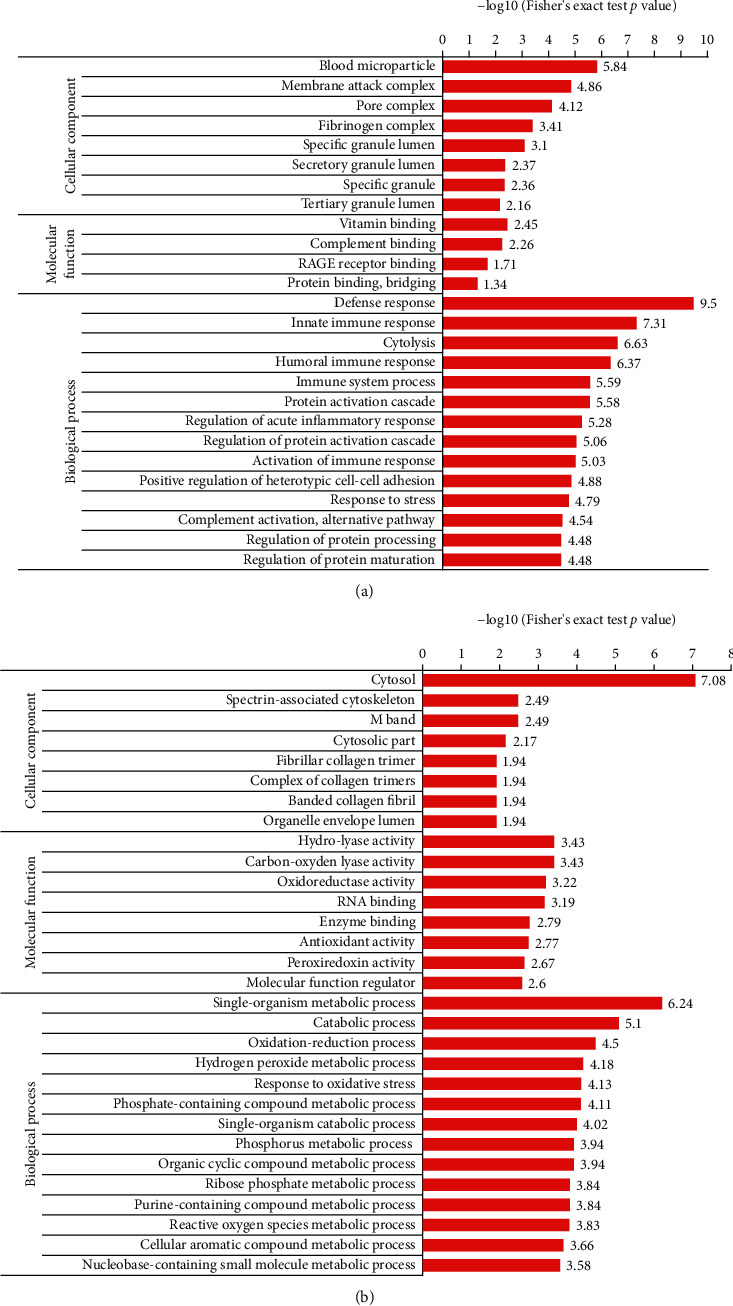
GO term analysis of differentially expressed proteins (DEPs): (a) GO terms of DEPs in the upregulated group; (b) GO terms of DEPs in the downregulated group. GO: Gene Ontology.

Above all, the significantly enriched GO terms of the biological process in the upregulated group were investigated. The top 12 GO terms were immune or inflammatory response related proteins, further demonstrating the result that significant enrichment of the immune or inflammatory response pathway was observed during the initiation and development process of epilepsy [8–11]. In addition, the top 3 GO terms of the molecular function in the upregulated group were vitamin binding, complement binding, or RAGE receptor binding related proteins, indicating significant influence of vitamin and immunoglobulin superfamily on epilepsy control [12–14]. The top 6 GO terms of the cellular component in the upregulated group were blood microparticle, membrane attack complex, pore complex, fibrinogen complex, and specific and secretory granule lumen related proteins, illustrating hyperactivation of the complement cascade and dysregulation of lipid and fibrinogen systems in epilepsy patients [15–21](Figure 1(a)). Then, the significantly enriched GO terms of the biological process in the downregulated group were investigated. The top 5 GO terms were oxidation-reduction and oxidative stress response related proteins, further demonstrating the important role of oxidative stress in epilepsy [22]. The top 7 GO terms of the molecular function in the downregulated group were oxidoreductase activity, antioxidant activity, and peroxiredoxin activity related proteins, indicating a significantly decreased level of antioxidant activity in the plasma of epilepsy patients. The top 4 GO terms of the cellular component in the downregulated group were cytosol and cytosolic part related proteins, illustrating that most of the active proteins are present in the cytoplasm (Figure 1(b)).

### 6.4. Enrichment Analysis of DEPs in the KEGG Pathway

**Figure 2:**
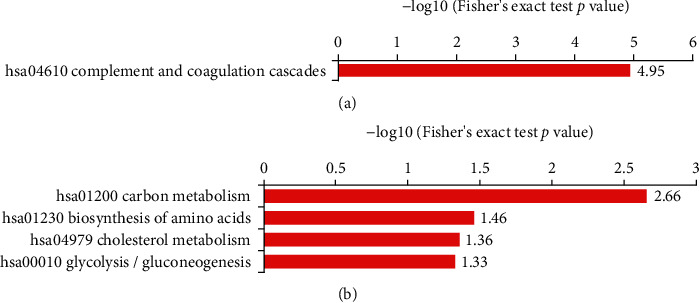
KEGG pathway enrichment analysis of DEPs: (a) pathway enrichment in the upregulated group; (b) pathway enrichment in the downregulated group. The pathway enrichment statistical analysis was performed by a two-tailed Fisher exact test. The *x*-axis is the –log10(*p* value). The *y*-axis is the enrichment pathway. KEGG: Kyoto Encyclopedia of Genes and Genomes.

KEGG pathway enrichment analysis demonstrated that the complement and coagulation cascades were the most significantly affected pathways in the upregulated group. In addition, various metabolic pathways were significantly enriched in the downregulated group, of which were carbon metabolism, biosynthesis of amino acids, cholesterol metabolism, and glycolysis pathways (Figure 2). The results of the KEGG pathway analysis further indicated that hyperactivation of the complement cascade and dysregulation of lipid and glycometabolism systems are associated with the process of epilepsy development.

### 6.5. PPI Coexpression Network and the Top 10 Hub Genes

**Figure 3:**
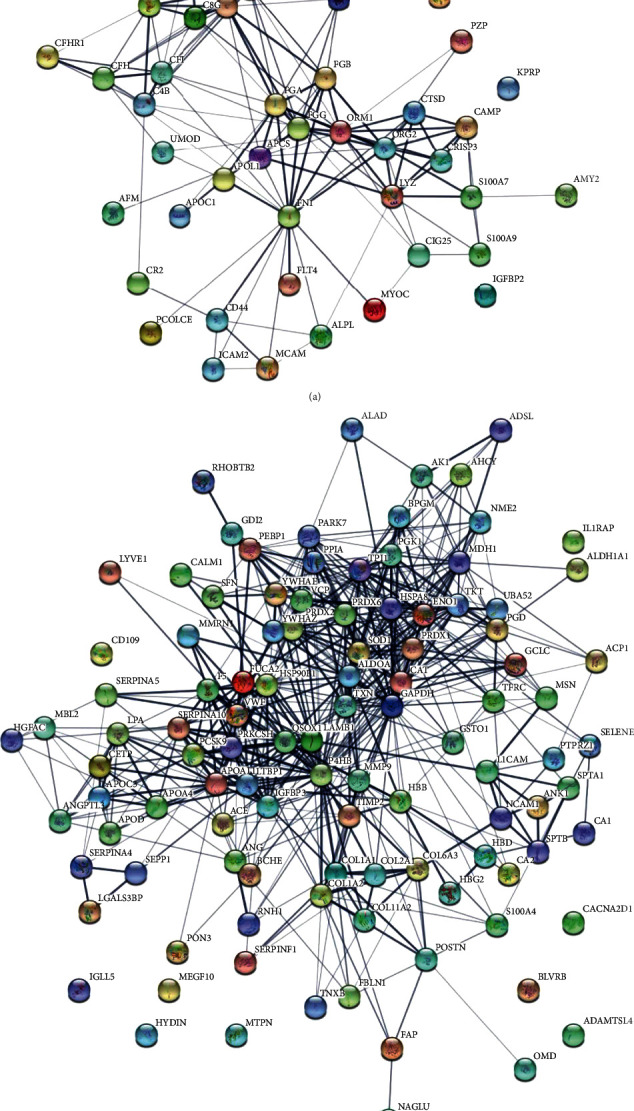
PPI coexpression network of DEPs: (a) PPI coexpression network in the upregulated group; (b) PPI coexpression network in the downregulated group.

The PPI networks of these DEPs in the up- and downregulated group are shown, respectively (Figure 3). Then, the top 10 hub genes were screened by cytoHubba according to the high degree of connectivity (Table 2). These hub genes were related with acute-phase or innate immune response, complement activation, regulation of fibrinogen systems, glycolysis, lipoprotein metabolism, and oxidative stress reaction.

## 7. Discussion

Rolandic epilepsy is an important and common disease in childhood. The cause of epilepsy remains unclear. In this study, we used a TMT-based quantitative proteomic analysis tool to detect the meaningful biomarkers in the plasma of Rolandic epilepsy patients. Here, we focus on hub genes in our study.

Fibronectin (FN1) was among the top 10 hub genes in the upregulated group which played an important role in maintaining cell morphology, intercellular adhesion, cell migration or chemotaxis, cell phagocytosis, hemostasis, damage repair, and body defense. Dixit et al. performed transcriptome analysis of hippocampal tissues resected from patients with MTLE-HS using the RNAseq approach. Differential gene expression analysis revealed that fibronectin was upregulated and was in the center of the network [23].

Alpha-1-acid glycoprotein (ORM) was also in the upregulated group which had many activities including, but not limited to, acting as an acute-phase reactant and disease marker, modulating immunity, binding and carrying drugs, maintaining the barrier function of capillary, and mediating the sphingolipid metabolism [24]. So far, there have been no reports on the relationship between alpha-1-acid glycoprotein and epilepsy.

Serum amyloid P-component (APCS) showed increase expression in the plasma of patients with epilepsy. Serum amyloid P-component belongs to the group of short pentraxins in the pentraxin family, which is known to be involved the humoral innate immune system spanning the complement system, inflammation, and coagulation [25]. Urbanyi et al. demonstrated that serum amyloid P-component induced neuronal apoptosis through penetrating the plasma membrane and translocating selectively into the nuclei of neurons [26]. But the relationship between serum amyloid P-component and epilepsy has not been known.

Fibrinogen (FGG, FGA) was also found to be increased in the plasma of patients with epilepsy. Hamed et al. reported that the level of fibrinogen was increased in the serum of adult patients with epilepsy compared with that of a healthy control, which demonstrated that vascular risk factor might be one of the independent predictors of asymptomatic atherosclerosis [27].

The complement system including complement component C9, complement component C8 beta chain, complement C4-B, and complement factor I was activated in the patient group, which was consistent with the result that the complement cascade was involved during epileptogenesis in the chronic epileptic phase in both experimental and human TLE [15]. The persistence of complement activation may contribute to a sustained inflammatory response and destabilize neuronal networks involved.

Some glycolytic enzymes including glyceraldehyde-3-phosphate dehydrogenase (GAPDH), triosephosphate isomerase (TPI1), phosphoglycerate kinase 1(PGK1), and fructose-bisphosphate aldolase A (ALDOA) play an important role in epileptogenesis. The decreased GAPDH kinase activity on *γ*-aminobutyric acid type A receptors (GABA_A_R) can reduce both endogenous phosphorylation and GABA_A_R function. This dysfunction likely contributes to seizure generation and/or transition from the interictal to the ictal state [28]. In addition to its important catalytic role in glycolysis, ALDOA has other functions, such as signal transduction, vesicle transport, and maintenance of cell activity. Interactions of aldolases A and C in light neurofilament (NF-L) expression may be linked to regulatory pathways that maintain the highly asymmetrical form and function of large neurons [29].

Some antioxidases such as catalase (CAT) and superoxide dismutase [Cu-Zn](SOD1) were in the downregulated group, which was consistent with the result found by Guler et al.'s research group [30]. Decreased activity of CAT and SOD1 in the plasma suggests a reduced antioxidant defense capacity in epilepsy patients. Protein disulfide-isomerase (P4HB), an endoplasmic reticulum chaperone, plays a crucial role in catalyzing disulfide bond formation, reduction, and isomerization. Kim et al. proved that protein disulfide-isomerase is bound to NMDAR in the normal hippocampus and that this binding was increased in chronic epileptic rats. In addition, protein disulfide-isomerase knockdown effectively ameliorated spontaneous seizure activity in chronic epileptic rats. The finding suggested that protein disulfide-isomerase might represent a target of potential therapeutics for epilepsy [31].

Heat shock protein 90 kDa beta member 1(HSP90B1), a member of the Hsp90 family, showed decrease expression in the plasma of patients with epilepsy. Hong et al. discovered that HSP90B1 was a critical chaperone to integrate innate immunity, Wnt signaling, and organ development [32]. Di et al. reported that repressing the initial ER-associated degradation recognition step by inhibiting HSP90B1 enhanced the functional surface expression of misfolding-prone *α*1(A322D) subunits of GABA_A_R, which caused autosomal dominant juvenile myoclonic epilepsy [33].

*α*-Enolase (ENO1) was also in the downregulated group. In addition to glycolytic enzyme and plasminogen receptor functions, *α*-enolase appears to have other cellular functions and subcellular localizations. As a plasminogen receptor, ENO1 binds to plasminogen and activates plasminogen conversion to plasmin. Ho-Tin-Noé et al. proved that limited plasminogen activation might contribute to nervous tissue organization during development, while uncontrolled plasmin formation might be neurotoxic [34]. Lahtinen et al. pointed out that the plasminogen system was involved in various events requiring extracellular proteolysis that were relevant to epileptogenesis, including cell migration and invasion, inflammation, and tissue repair and remodelling [35].

Apolipoprotein A1 (APOA1) was also found to be decreased in the plasma of patients with epilepsy. Apolipoprotein A1, a major component of high-density lipoproteins, has been shown to be involved in lipid metabolism, cholesterol homeostasis, and degeneration/regeneration of brain tissues and is proposed as a useful marker for the extent and severity of CNS injury. Ni et al. reported that in the model of neonatal SD rats induced by inhalation of flurothyl, the expression level of apolipoprotein A1 mRNA in the hippocampus of the recurrent epilepsy group was significantly decreased 30 days after the first seizure compared with the control group [36]. We speculate that the decreased level of apolipoprotein A1 in patients with epilepsy may indicate abnormal lipid metabolism and high risk of atherosclerosis.

However, there are some limitations of the study, such as the limited sample size. We have only included the minimum number of biological repeats required for proteomic analysis. But our study may provide clues for further study of large samples.

## 8. Conclusions

Our findings demonstrate that some differentially expressed proteins especially hub genes identified by TMT-based proteomics combined with bioinformatics analysis may be associated with epileptogenesis. Activation of acute-phase or innate immune response, complement and fibrinogen systems, and repression of glycolysis, lipoprotein metabolism, and antioxidant activity may play a role in the development of epilepsy. Some novel proteins are found out such as alpha-1-acid glycoprotein (ORM) and serum amyloid P-component (APCS). These new findings may provide novel insights into the pathogenic mechanisms of epilepsy and lead to a new therapeutic method in the future.

## Figures and Tables

**Table 1:** Clinical features of patients with Rolandic epilepsy and controls.

Number	Sex	Age (year)	Age of onset (year)	Duration (years)	VEEG/EEG
Patients with Rolandic epilepsy					
E1	M	9.8 y	5	4	SP (B)
E2	M	11 y	10	0.3	SP (R)
E3	M	7.4 y	6	1	SP (L)
E4	F	9 y	8	1	SP (R)
E5	F	9.2 y	8	1	SP (B)
Number	Sex	Age (year)	Duration (years)	MRI	VEEG/EEG
Controls					
C1	M	10 y	5	n	n
C2	M	11 y	7	n	n
C3	M	9 y	3	n	n
C4	F	9 y	3	n	n
C5	F	10 y	4	n	n

M: male; F: female; R: right; L: left; B: Bilateral; EEG: electroencephalogram; VEEG: video electroencephalogram; SP: spike wave; n: normal; MRI: magnetic resonance imaging. E1–E5 represented five patients with Rolandic epilepsy; C1–C5 represented five patients with migraine.

**Table 2:** Top 10 hub genes in the upregulated group as well as top 10 hub genes in the downregulated group.

Top 10 hub genes in upregulated group			Top 10 hub genes in the downregulated group		
Database identifier	Display name	Degree	Database identifier	Display name	Degree
9606.ENSP00000346839	FN1	17	9606.ENSP00000229239	GAPDH	47
9606.ENSP00000259396	ORM1	16	9606.ENSP00000327801	P4HB	36
9606.ENSP00000394936	ORM2	14	9606.ENSP00000234590	ENO1	30
9606.ENSP00000336829	FGG	13	9606.ENSP00000229270	TPI1	27
9606.ENSP00000306361	FGA	13	9606.ENSP00000362413	PGK1	25
9606.ENSP00000263408	C9	12	9606.ENSP00000236850	APOA1	25
9606.ENSP00000360281	C8B	12	9606.ENSP00000241052	CAT	23
9606.ENSP00000255040	APCS	10	9606.ENSP00000270142	SOD1	23
9606.ENSP00000415941	C4B	10	9606.ENSP00000378669	ALDOA	22
9606.ENSP00000378130	CFI	10	9606.ENSP00000299767	HSP90B1	22

## Data Availability

All relevant data are within the paper and its Supporting Information files.

## Supplementary Materials

Supplementary 1 Table S1: MS/MS spectrum database search analysis summary.

Supplementary 2 Figure S1: experimental strategy for quantitative proteome analysis and quality control validation of MS data. (a) Average peptide mass error. (b) Length distribution of all identified peptides.

Supplementary 3 Table S2: the list of identified protein between the epilepsy and control groups by TMT-based proteomics analysis.

Supplementary 4 Table S3: the list of differentially expressed protein between the epilepsy and control groups by TMT-based proteomics analysis.
