# Supplementary material for: TMT-Based Proteomic Analysis of Plasma from Children with Rolandic Epilepsy
Source: Dis Markers. 2020 Oct 7;2020:8840482. doi: 10.1155/2020/8840482 (PMC7563079; doi:10.1155/2020/8840482)
Supplement: Supplementary 1 — Table S1: MS/MS spectrum database search analysis summary. [file 8840482.f1.pdf]

TABLE S1. MS/MS spectrum database search analysis summary

Total spectrum	Matched spectrum	Peptides	Unique peptides	Identified proteins	Quantifiable proteins
213300	17617	4659	4541	752	670
