# Supplementary figures and images for: TMT-Based Proteomic Analysis of Plasma from Children with Rolandic Epilepsy
Source: Dis Markers. 2020 Oct 7;2020:8840482. doi: 10.1155/2020/8840482 (PMC7563079; doi:10.1155/2020/8840482)

a

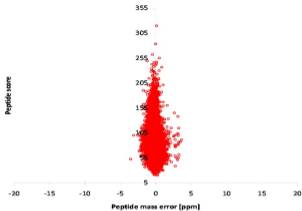

b

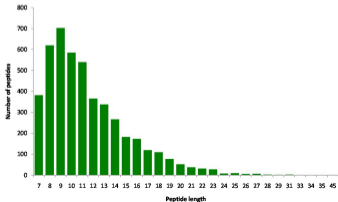

Supplement: Supplementary 2 — Figure S1: experimental strategy for quantitative proteome analysis and quality control validation of MS data. (a) Average peptide mass error. (b) Length distribution of all identified peptides. [file 8840482.f2.pdf]
