# Supplementary material for: TMT-Based Proteomic Analysis of Plasma from Children with Rolandic Epilepsy
Source: Dis Markers. 2020 Oct 7;2020:8840482. doi: 10.1155/2020/8840482 (PMC7563079; doi:10.1155/2020/8840482)
Supplement: Supplementary 3 — Table S2: the list of identified protein between the epilepsy and control groups by TMT-based proteomics analysis. [file 8840482.f3.pdf]

Table S2.The list of identified protein between epilepsy and control group by TMT-based proteomics analysis.

Protein accession	Protein description	Gene name	MW [kDa]	Score	Peptides
A0A075B6S6	Immunoglobulin kappa variable 2D-30 OS=Homo sapiens GN=IGKV2D-30	IGKV2D-30	13.215	26.129	1
A0JNW5	UHRF1-binding protein 1-like OS=Homo sapiens GN=UHRF1BP1L	UHRF1BP1L	164.2	-2	1
A1L4H1	Soluble scavenger receptor cysteine-rich domain-containing protein SSC5D OS=Homo sapiens GN=SSC5D	SSC5D	165.74	63.166	9
A6NCI4	von Willebrand factor A domain-containing protein 3A OS=Homo sapiens GN=VWA3A	VWA3A	134.02	6.4567	1
B9A064	Immunoglobulin lambda-like polypeptide 5 OS=Homo sapiens GN=IGLL5	IGLL5	23.063	88.627	5
I6L899	Golgin subfamily A member 8R OS=Homo sapiens GN=GOLGA8R	GOLGA8R	71.489	-2	1
O00142	Thymidine kinase 2, mitochondrial OS=Homo sapiens GN=TK2	TK2	31.004	7.0125	1
O00187	Mannan-binding lectin serine protease 2 OS=Homo sapiens GN=MASP2	MASP2	75.702	192.91	12
O00299	Chloride intracellular channel protein 1 OS=Homo sapiens GN=CLIC1	CLIC1	26.922	6.3136	1
O00391	Sulfhydryl oxidase 1 OS=Homo sapiens GN=QSOX1	QSOX1	82.577	177.41	13
O00429	Dynamin-1-like protein OS=Homo sapiens GN=DNM1L	DNM1L	81.876	7.7284	1
O00462	Beta-mannosidase OS=Homo sapiens GN=MANBA	MANBA	100.89	20.882	3
O00533	Neural cell adhesion molecule L1-like protein OS=Homo sapiens GN=CHL1	CHL1	135.07	318.4	17
O00592	Podocalyxin OS=Homo sapiens GN=PODXL	PODXL	58.635	19.174	2
O00602	Ficolin-1 OS=Homo sapiens GN=FCN1	FCN1	35.078	26.434	2
O14498	Immunoglobulin superfamily containing leucine-rich repeat protein OS=Homo sapiens GN=ISLR	ISLR	45.997	12.563	2
O14786	Neuropilin-1 OS=Homo sapiens GN=NRP1	NRP1	103.13	153.59	6
O14791	Apolipoprotein L1 OS=Homo sapiens GN=APOL1	APOL1	43.974	187.11	15
O14810	Complexin-1 OS=Homo sapiens GN=CPLX1	CPLX1	15.03	50.144	1
O14983	Sarcoplasmic/endoplasmic reticulum calcium ATPase 1 OS=Homo sapiens GN=ATP2A1	ATP2A1	110.25	14.293	1
O15031	Plexin-B2 OS=Homo sapiens GN=PLXNB2	PLXNB2	205.12	62.488	3
O15061	Synemin OS=Homo sapiens GN=SYNM	SYNM	172.77	6.7284	1
O15335	Chondroadherin OS=Homo sapiens GN=CHAD	CHAD	40.475	45.321	5
O15394	Neural cell adhesion molecule 2 OS=Homo sapiens GN=NCAM2	NCAM2	93.045	67.499	9
O43493	Trans-Golgi network integral membrane protein 2 OS=Homo sapiens GN=TGOLN2	TGOLN2	51.112	35.819	5
O43505	Beta-1,4-glucuronyltransferase 1 OS=Homo sapiens GN=B4GAT1	B4GAT1	47.119	43.726	3
O43866	CD5 antigen-like OS=Homo sapiens GN=CD5L	CD5L	38.087	105.39	10
O60234	Glia maturation factor gamma OS=Homo sapiens GN=GMFG	GMFG	16.801	13.225	2
O60462	Neuropilin-2 OS=Homo sapiens GN=NRP2	NRP2	104.86	64.358	6
O60641	Clathrin coat assembly protein AP180 OS=Homo sapiens GN=SNAP91	SNAP91	92.501	23.207	3
O60832	H/ACA ribonucleoprotein complex subunit 4 OS=Homo sapiens GN=DKC1	DKC1	57.673	6.4304	1
O75144	ICOS ligand OS=Homo sapiens GN=ICOSLG	ICOSLG	33.348	38.771	2
O75356	Ectonucleoside triphosphate diphosphohydrolase 5 OS=Homo sapiens GN=ENTPD5	ENTPD5	47.517	6.2133	1
O75636	Ficolin-3 OS=Homo sapiens GN=FCN3	FCN3	32.903	68.074	7
O75882	Attractin OS=Homo sapiens GN=ATRN	ATRN	158.54	323.31	34
O75891	Cytosolic 10-formyltetrahydrofolate dehydrogenase OS=Homo sapiens GN=ALDH1L1	ALDH1L1	98.828	7.1455	1
O94769	Extracellular matrix protein 2 OS=Homo sapiens GN=ECM2	ECM2	79.788	7.0901	1
O95274	Ly6/PLAUR domain-containing protein 3 OS=Homo sapiens GN=LYPD3	LYPD3	35.97	6.2181	1
O95428	Papilin OS=Homo sapiens GN=PAPLN	PAPLN	137.7	24.535	3
O95445	Apolipoprotein M OS=Homo sapiens GN=APOM	APOM	21.253	56.881	3
O95479	GDH/6PGL endoplasmic bifunctional protein OS=Homo sapiens GN=H6PD	H6PD	88.892	67.667	8
O95497	Pantetheinase OS=Homo sapiens GN=VNN1	VNN1	57.011	14.966	1
O95563	Mitochondrial pyruvate carrier 2 OS=Homo sapiens GN=MPC2	MPC2	14.279	14.461	1
O95670	V-type proton ATPase subunit G 2 OS=Homo sapiens GN=ATP6V1G2	ATP6V1G2	13.604	34.516	1
O95897	Noelin-2 OS=Homo sapiens GN=OLFM2	OLFM2	51.386	7.466	1
O95967	EGF-containing fibulin-like extracellular matrix protein 2 OS=Homo sapiens GN=EFEMP2	EFEMP2	49.405	34.197	2
O95980	Reversion-inducing cysteine-rich protein with Kazal motifs OS=Homo sapiens GN=RECK	RECK	106.46	16.874	2
O95998	Interleukin-18-binding protein OS=Homo sapiens GN=IL18BP	IL18BP	21.099	13.751	2
P00167	Cytochrome b5 OS=Homo sapiens GN=CYB5A	CYB5A	15.33	7.0425	1
P00325	Alcohol dehydrogenase 1B OS=Homo sapiens GN=ADH1B	ADH1B	39.854	18.944	3
P00338	L-lactate dehydrogenase A chain OS=Homo sapiens GN=LDHA	LDHA	36.688	63.298	6
P00352	Retinal dehydrogenase 1 OS=Homo sapiens GN=ALDH1A1	ALDH1A1	54.861	32.536	4
P00441	Superoxide dismutase [Cu-Zn] OS=Homo sapiens GN=SOD1	SOD1	15.936	33.18	2
P00450	Ceruloplasmin OS=Homo sapiens GN=CP	CP	122.2	323.31	38
P00451	Coagulation factor VIII OS=Homo sapiens GN=F8	F8	267.01	23.248	4
P00488	Coagulation factor XIII A chain OS=Homo sapiens GN=F13A1	F13A1	83.266	85.568	8
P00491	Purine nucleoside phosphorylase OS=Homo sapiens GN=PNP	PNP	32.118	36.119	4
P00492	Hypoxanthine-guanine phosphoribosyltransferase OS=Homo sapiens GN=HPRT1	HPRT1	24.579	19.136	2
P00533	Epidermal growth factor receptor OS=Homo sapiens GN=EGFR	EGFR	134.28	17.916	3
P00558	Phosphoglycerate kinase 1 OS=Homo sapiens GN=PGK1	PGK1	44.614	136.66	11
P00568	Adenylate kinase isoenzyme 1 OS=Homo sapiens GN=AK1	AK1	21.635	26.423	3
P00709	Alpha-lactalbumin OS=Homo sapiens GN=LALBA	LALBA	16.225	6.3777	1
P00734	Prothrombin OS=Homo sapiens GN=F2	F2	70.036	323.31	31
P00736	Complement C1r subcomponent OS=Homo sapiens GN=C1R	C1R	80.118	323.31	21
P00738	Haptoglobin OS=Homo sapiens GN=HP	HP	45.205	323.31	22
P00740	Coagulation factor IX OS=Homo sapiens GN=F9	F9	51.778	323.31	10
P00742	Coagulation factor X OS=Homo sapiens GN=F10	F10	54.731	323.31	15
P00746	Complement factor D OS=Homo sapiens GN=CFD	CFD	27.033	132.17	3
P00747	Plasminogen OS=Homo sapiens GN=PLG	PLG	90.568	323.31	41
P00748	Coagulation factor XII OS=Homo sapiens GN=F12	F12	67.791	210.25	10
P00751	Complement factor B OS=Homo sapiens GN=CFB	CFB	85.532	323.31	33
P00915	Carbonic anhydrase 1 OS=Homo sapiens GN=CA1	CA1	28.87	312.95	8
P00918	Carbonic anhydrase 2 OS=Homo sapiens GN=CA2	CA2	29.246	100.07	6
P01008	Antithrombin-III OS=Homo sapiens GN=SERPINC1	SERPINC1	52.602	323.31	19
P01009	Alpha-1-antitrypsin OS=Homo sapiens GN=SERPINA1	SERPINA1	46.736	323.31	14
P01011	Alpha-1-antichymotrypsin OS=Homo sapiens GN=SERPINA3	SERPINA3	47.65	323.31	13
P01019	Angiotensinogen OS=Homo sapiens GN=AGT	AGT	53.154	208.15	7
P01023	Alpha-2-macroglobulin OS=Homo sapiens GN=A2M	A2M	163.29	323.31	44
P01024	Complement C3 OS=Homo sapiens GN=C3	C3	187.15	323.31	90
P01031	Complement C5 OS=Homo sapiens GN=C5	C5	188.3	323.31	52
P01033	Metalloproteinase inhibitor 1 OS=Homo sapiens GN=TIMP1	TIMP1	23.171	22.044	3
P01034	Cystatin-C OS=Homo sapiens GN=CST3	CST3	15.799	155.25	5
P01040	Cystatin-A OS=Homo sapiens GN=CSTA	CSTA	11.006	7.7357	1
P01042	Kininogen-1 OS=Homo sapiens GN=KNG1	KNG1	71.957	323.31	28
P01116	GTPase KRas OS=Homo sapiens GN=KRAS	KRAS	21.656	17.711	1
P01130	Low-density lipoprotein receptor OS=Homo sapiens GN=LDLR	LDLR	95.375	6.2537	1
P01137	Transforming growth factor beta-1 OS=Homo sapiens GN=TGFB1	TGFB1	44.341	7.2306	1

P01344	Insulin-like growth factor II OS=Homo sapiens GN=IGF2	IGF2	20.14	54.22	3
P01591	Immunoglobulin J chain OS=Homo sapiens GN=JCHAIN	JCHAIN	18.098	29.367	3
P01602	Immunoglobulin kappa variable 1-5 OS=Homo sapiens GN=IGKV1-5	IGKV1-5	12.781	6.9311	1
P01701	Immunoglobulin lambda variable 1-51 OS=Homo sapiens GN=IGLV1-51	IGLV1-51	12.249	12.199	2
P01714	Immunoglobulin lambda variable 3-19 OS=Homo sapiens GN=IGLV3-19	IGLV3-19	12.042	12.658	2
P01780	Immunoglobulin heavy variable 3-7 OS=Homo sapiens GN=IGHV3-7	IGHV3-7	12.943	17.271	2
P01834	Ig kappa chain C region OS=Homo sapiens GN=IGKC	IGKC	11.609	205.04	3
P01857	Ig gamma-1 chain C region OS=Homo sapiens GN=IGHG1	IGHG1	36.105	226.55	10
P01859	Ig gamma-2 chain C region OS=Homo sapiens GN=IGHG2	IGHG2	35.9	6.2103	6
P01860	Ig gamma-3 chain C region OS=Homo sapiens GN=IGHG3	IGHG3	41.287	52.871	9
P01861	Ig gamma-4 chain C region OS=Homo sapiens GN=IGHG4	IGHG4	35.94	57.237	6
P01871	Ig mu chain C region OS=Homo sapiens GN=IGHM	IGHM	49.306	323.31	15
P01876	Ig alpha-1 chain C region OS=Homo sapiens GN=IGHA1	IGHA1	37.654	164.99	7
P01877	Ig alpha-2 chain C region OS=Homo sapiens GN=IGHA2	IGHA2	36.526	21.457	6
P02042	Hemoglobin subunit delta OS=Homo sapiens GN=HBD	HBD	16.055	96.689	10
P02144	Myoglobin OS=Homo sapiens GN=MB	MB	17.184	7.3267	1
P02452	Collagen alpha-1(I) chain OS=Homo sapiens GN=COL1A1	COL1A1	138.94	96.393	10
P02458	Collagen alpha-1(II) chain OS=Homo sapiens GN=COL2A1	COL2A1	141.78	73.199	5
P02549	Spectrin alpha chain, erythrocytic 1 OS=Homo sapiens GN=SPTA1	SPTA1	280.01	121.51	13
P02647	Apolipoprotein A-I OS=Homo sapiens GN=APOA1	APOA1	30.777	323.31	24
P02649	Apolipoprotein E OS=Homo sapiens GN=APOE	APOE	36.154	323.31	20
P02652	Apolipoprotein A-II OS=Homo sapiens GN=APOA2	APOA2	11.175	32.979	4
P02654	Apolipoprotein C-I OS=Homo sapiens GN=APOC1	APOC1	9.3318	61.452	5
P02655	Apolipoprotein C-II OS=Homo sapiens GN=APOC2	APOC2	11.284	32.549	3
P02656	Apolipoprotein C-III OS=Homo sapiens GN=APOC3	APOC3	10.852	186.4	3
P02671	Fibrinogen alpha chain OS=Homo sapiens GN=FGA	FGA	94.972	323.31	28
P02675	Fibrinogen beta chain OS=Homo sapiens GN=FGB	FGB	55.928	323.31	27
P02679	Fibrinogen gamma chain OS=Homo sapiens GN=FGG	FGG	51.511	323.31	19
P02730	Band 3 anion transport protein OS=Homo sapiens GN=SLC4A1	SLC4A1	101.79	32.374	5
P02741	C-reactive protein OS=Homo sapiens GN=CRP	CRP	25.038	7.3268	1
P02743	Serum amyloid P-component OS=Homo sapiens GN=APCS	APCS	25.387	112.88	6
P02745	Complement C1q subcomponent subunit A OS=Homo sapiens GN=C1QA	C1QA	26.016	20.216	2
P02746	Complement C1q subcomponent subunit B OS=Homo sapiens GN=C1QB	C1QB	26.721	137.41	3
P02747	Complement C1q subcomponent subunit C OS=Homo sapiens GN=C1QC	C1QC	25.773	173.92	4
P02748	Complement component C9 OS=Homo sapiens GN=C9	C9	63.173	323.31	16
P02749	Beta-2-glycoprotein 1 OS=Homo sapiens GN=APOH	APOH	38.298	323.31	19
P02750	Leucine-rich alpha-2-glycoprotein OS=Homo sapiens GN=LRG1	LRG1	38.177	149.1	8
P02751	Fibronectin OS=Homo sapiens GN=FN1	FN1	262.62	323.31	34
P02753	Retinol-binding protein 4 OS=Homo sapiens GN=RBP4	RBP4	23.01	75.542	4
P02760	Protein AMBP OS=Homo sapiens GN=AMBP	AMBP	38.999	323.31	12
P02763	Alpha-1-acid glycoprotein 1 OS=Homo sapiens GN=ORM1	ORM1	23.511	98.287	6
P02765	Alpha-2-HS-glycoprotein OS=Homo sapiens GN=AHSG	AHSG	39.324	323.31	9
P02766	Transthyretin OS=Homo sapiens GN=TTR	TTR	15.887	87.643	3
P02774	Vitamin D-binding protein OS=Homo sapiens GN=GC	GC	52.963	323.31	25
P02775	Platelet basic protein OS=Homo sapiens GN=PPBP	PPBP	13.894	50.371	6
P02776	Platelet factor 4 OS=Homo sapiens GN=PF4	PF4	10.845	8.2302	3
P02786	Transferrin receptor protein 1 OS=Homo sapiens GN=TFRC	TFRC	84.87	62.244	9
P02787	Serotransferrin OS=Homo sapiens GN=TF	TF	77.063	323.31	34
P02788	Lactotransferrin OS=Homo sapiens GN=LTF	LTF	78.181	48.91	4
P02790	Hemopexin OS=Homo sapiens GN=HPX	HPX	51.676	323.31	21
P02810	Salivary acidic proline-rich phosphoprotein 1/2 OS=Homo sapiens GN=PRH1	PRH1	17.016	8.3196	1
P03950	Angiogenin OS=Homo sapiens GN=ANG	ANG	16.55	21.691	3
P03951	Coagulation factor XI OS=Homo sapiens GN=F11	F11	70.108	301.9	20
P03952	Plasma kallikrein OS=Homo sapiens GN=KLKB1	KLKB1	71.369	323.31	29
P03973	Antileukoproteinase OS=Homo sapiens GN=SLPI	SLPI	14.326	7.1151	1
P04003	C4b-binding protein alpha chain OS=Homo sapiens GN=C4BPA	C4BPA	67.033	323.31	22
P04004	Vitronectin OS=Homo sapiens GN=VTN	VTN	54.305	323.31	18
P04040	Catalase OS=Homo sapiens GN=CAT	CAT	59.755	267.16	17
P04066	Tissue alpha-L-fucosidase OS=Homo sapiens GN=FUCA1	FUCA1	53.688	10.647	2
P04070	Vitamin K-dependent protein C OS=Homo sapiens GN=PROC	PROC	52.071	48.781	6
P04075	Fructose-bisphosphate aldolase A OS=Homo sapiens GN=ALDOA	ALDOA	39.42	219.47	9
P04114	Apolipoprotein B-100 OS=Homo sapiens GN=APOB	APOB	515.6	323.31	182
P04180	Phosphatidylcholine-sterol acyltransferase OS=Homo sapiens GN=LCAT	LCAT	49.577	90.021	7
P04196	Histidine-rich glycoprotein OS=Homo sapiens GN=HRG	HRG	59.578	323.31	16
P04217	Alpha-1B-glycoprotein OS=Homo sapiens GN=A1BG	A1BG	54.253	226.94	9
P04275	von Willebrand factor OS=Homo sapiens GN=VWF	VWF	309.26	323.31	56
P04278	Sex hormone-binding globulin OS=Homo sapiens GN=SHBG	SHBG	43.779	108.69	7
P04406	Glyceraldehyde-3-phosphate dehydrogenase OS=Homo sapiens GN=GAPDH	GAPDH	36.053	82.064	6
P04424	Argininosuccinate lyase OS=Homo sapiens GN=ASL	ASL	51.657	9.7413	1
P04433	Immunoglobulin kappa variable 3-11 OS=Homo sapiens GN=IGKV3-11	IGKV3-11	12.575	6.2181	1
P04792	Heat shock protein beta-1 OS=Homo sapiens GN=HSPB1	HSPB1	22.782	8.7538	1
P05019	Insulin-like growth factor I OS=Homo sapiens GN=IGF1	IGF1	21.841	6.9388	1
P05060	Secretogranin-1 OS=Homo sapiens GN=CHGB	CHGB	78.275	8.107	1
P05062	Fructose-bisphosphate aldolase B OS=Homo sapiens GN=ALDOB	ALDOB	39.473	63.903	8
P05067	Amyloid beta A4 protein OS=Homo sapiens GN=APP	APP	86.942	21.046	2
P05089	Arginase-1 OS=Homo sapiens GN=ARG1	ARG1	34.735	13.365	2
P05090	Apolipoprotein D OS=Homo sapiens GN=APOD	APOD	21.275	123.67	6
P05106	Integrin beta-3 OS=Homo sapiens GN=ITGB3	ITGB3	87.057	15.725	2
P05107	Integrin beta-2 OS=Homo sapiens GN=ITGB2	ITGB2	84.781	11.218	2
P05109	Protein S100-A8 OS=Homo sapiens GN=S100A8	S100A8	10.834	36.96	5
P05154	Plasma serine protease inhibitor OS=Homo sapiens GN=SERPINA5	SERPINA5	45.674	203.92	11
P05155	Plasma protease C1 inhibitor OS=Homo sapiens GN=SERPING1	SERPING1	55.154	323.31	13
P05156	Complement factor I OS=Homo sapiens GN=CFI	CFI	65.75	323.31	16
P05164	Myeloperoxidase OS=Homo sapiens GN=MPO	MPO	83.868	11.391	2
P05186	Alkaline phosphatase, tissue-nonspecific isozyme OS=Homo sapiens GN=ALPL	ALPL	57.304	46.479	3
P05362	Intercellular adhesion molecule 1 OS=Homo sapiens GN=ICAM1	ICAM1	57.825	174.69	9
P05451	Lithostathine-1-alpha OS=Homo sapiens GN=REG1A	REG1A	18.731	11.552	2
P05543	Thyroxine-binding globulin OS=Homo sapiens GN=SERPINA7	SERPINA7	46.324	263.29	9
P05546	Heparin cofactor 2 OS=Homo sapiens GN=SERPIND1	SERPIND1	57.07	228.44	14

P05556	Integrin beta-1 OS=Homo sapiens GN=ITGB1	ITGB1	88.414	82.705	9
P05997	Collagen alpha-2(V) chain OS=Homo sapiens GN=COL5A2	COL5A2	144.91	6.7274	2
P06276	Cholinesterase OS=Homo sapiens GN=BCHE	BCHE	68.417	118.5	9
P06312	Immunoglobulin kappa variable 4-1 OS=Homo sapiens GN=IGKV4-1	IGKV4-1	13.38	7.1605	1
P06576	ATP synthase subunit beta, mitochondrial OS=Homo sapiens GN=ATP5B	ATP5B	56.559	22.288	1
P06681	Complement C2 OS=Homo sapiens GN=C2	C2	83.267	323.31	23
P06702	Protein S100-A9 OS=Homo sapiens GN=S100A9	S100A9	13.242	62.968	6
P06727	Apolipoprotein A-IV OS=Homo sapiens GN=APOA4	APOA4	45.398	323.31	33
P06732	Creatine kinase M-type OS=Homo sapiens GN=CKM	CKM	43.101	31.829	5
P06733	Alpha-enolase OS=Homo sapiens GN=ENO1	ENO1	47.168	69.109	8
P06753	Tropomyosin alpha-3 chain OS=Homo sapiens GN=TPM3	TPM3	32.95	23.138	3
P07148	Fatty acid-binding protein, liver OS=Homo sapiens GN=FABP1	FABP1	14.208	16.046	2
P07195	L-lactate dehydrogenase B chain OS=Homo sapiens GN=LDHB	LDHB	36.638	20.779	4
P07196	Neurofilament light polypeptide OS=Homo sapiens GN=NEFL	NEFL	61.516	98.132	1
P07225	Vitamin K-dependent protein S OS=Homo sapiens GN=PROS1	PROS1	75.122	323.31	20
P07237	Protein disulfide-isomerase OS=Homo sapiens GN=P4HB	P4HB	57.116	52.312	7
P07307	Asialoglycoprotein receptor 2 OS=Homo sapiens GN=ASGR2	ASGR2	35.092	11.015	2
P07327	Alcohol dehydrogenase 1A OS=Homo sapiens GN=ADH1A	ADH1A	39.858	6.2103	3
P07333	Macrophage colony-stimulating factor 1 receptor OS=Homo sapiens GN=CSF1R	CSF1R	107.98	35.907	3
P07339	Cathepsin D OS=Homo sapiens GN=CTSD	CTSD	44.552	93.044	7
P07355	Annexin A2 OS=Homo sapiens GN=ANXA2	ANXA2	38.604	11.004	2
P07357	Complement component C8 alpha chain OS=Homo sapiens GN=C8A	C8A	65.163	323.31	18
P07358	Complement component C8 beta chain OS=Homo sapiens GN=C8B	C8B	67.046	292.77	18
P07359	Platelet glycoprotein Ib alpha chain OS=Homo sapiens GN=GP1BA	GP1BA	71.539	110.65	5
P07360	Complement component C8 gamma chain OS=Homo sapiens GN=C8G	C8G	22.277	240.2	9
P07451	Carbonic anhydrase 3 OS=Homo sapiens GN=CA3	CA3	29.557	14.031	1
P07476	Involucrin OS=Homo sapiens GN=IVL	IVL	68.478	6.6416	1
P07585	Decorin OS=Homo sapiens GN=DCN	DCN	39.746	11.836	2
P07602	Prosaposin OS=Homo sapiens GN=PSAP	PSAP	58.112	42.48	7
P07686	Beta-hexosaminidase subunit beta OS=Homo sapiens GN=HEXB	HEXB	63.111	13.224	2
P07711	Cathepsin L1 OS=Homo sapiens GN=CTSL	CTSL	37.564	8.9293	1
P07737	Profilin-1 OS=Homo sapiens GN=PFN1	PFN1	15.054	58.58	5
P07738	Bisphosphoglycerate mutase OS=Homo sapiens GN=BPGM	BPGM	30.005	53.886	5
P07858	Cathepsin B OS=Homo sapiens GN=CTSB	CTSB	37.821	18.814	3
P07900	Heat shock protein HSP 90-alpha OS=Homo sapiens GN=HSP90AA1	HSP90AA1	84.659	63.321	6
P07911	Uromodulin OS=Homo sapiens GN=UMOD	UMOD	69.76	27.03	4
P07942	Laminin subunit beta-1 OS=Homo sapiens GN=LAMB1	LAMB1	198.04	112.48	13
P08123	Collagen alpha-2(I) chain OS=Homo sapiens GN=COL1A2	COL1A2	129.31	44.432	4
P08174	Complement decay-accelerating factor OS=Homo sapiens GN=CD55	CD55	41.4	50.408	7
P08185	Corticosteroid-binding globulin OS=Homo sapiens GN=SERPINA6	SERPINA6	45.14	237.72	7
P08195	4F2 cell-surface antigen heavy chain OS=Homo sapiens GN=SLC3A2	SLC3A2	67.993	131.02	9
P08253	72 kDa type IV collagenase OS=Homo sapiens GN=MMP2	MMP2	73.881	103.61	10
P08294	Extracellular superoxide dismutase [Cu-Zn] OS=Homo sapiens GN=SOD3	SOD3	25.851	79.496	4
P08493	Matrix Gla protein OS=Homo sapiens GN=MGP	MGP	12.353	10.86	1
P08514	Integrin alpha-IIb OS=Homo sapiens GN=ITGA2B	ITGA2B	113.38	6.6313	1
P08519	Apolipoprotein(a) OS=Homo sapiens GN=LPA	LPA	501.31	323.31	18
P08571	Monocyte differentiation antigen CD14 OS=Homo sapiens GN=CD14	CD14	40.076	190.59	8
P08581	Hepatocyte growth factor receptor OS=Homo sapiens GN=MET	MET	155.54	26.86	4
P08603	Complement factor H OS=Homo sapiens GN=CFH	CFH	139.09	323.31	52
P08637	Low affinity immunoglobulin gamma Fc region receptor III-A OS=Homo sapiens GN=FCGR3A	FCGR3A	29.089	20.366	3
P08697	Alpha-2-antiplasmin OS=Homo sapiens GN=SERPINF2	SERPINF2	54.565	323.31	17
P08709	Coagulation factor VII OS=Homo sapiens GN=F7	F7	51.593	24.817	3
P08887	Interleukin-6 receptor subunit alpha OS=Homo sapiens GN=IL6R	IL6R	51.547	12.599	2
P09172	Dopamine beta-hydroxylase OS=Homo sapiens GN=DBH	DBH	69.064	101.46	7
P09211	Glutathione S-transferase P OS=Homo sapiens GN=GSTP1	GSTP1	23.356	11.575	2
P09382	Galectin-1 OS=Homo sapiens GN=LGALS1	LGALS1	14.716	11.92	2
P09486	SPARC OS=Homo sapiens GN=SPARC	SPARC	34.632	93.372	7
P09603	Macrophage colony-stimulating factor 1 OS=Homo sapiens GN=CSF1	CSF1	60.179	7.1623	1
P09668	Pro-cathepsin H OS=Homo sapiens GN=CTSH	CTSH	37.393	13.83	2
P09681	Gastric inhibitory polypeptide OS=Homo sapiens GN=GIP	GIP	17.107	7.0598	1
P09871	Complement C1s subcomponent OS=Homo sapiens GN=C1S	C1S	76.684	323.31	17
P09960	Leukotriene A-4 hydrolase OS=Homo sapiens GN=LTA4H	LTA4H	69.284	86.211	7
P09972	Fructose-bisphosphate aldolase C OS=Homo sapiens GN=ALDOC	ALDOC	39.455	93.839	3
P0C0L4	Complement C4-A OS=Homo sapiens GN=C4A	C4A	192.78	323.31	64
P0C0L5	Complement C4-B OS=Homo sapiens GN=C4B	C4B	192.75	82.739	62
P0CG06	Ig lambda-3 chain C regions OS=Homo sapiens GN=IGLC3	IGLC3	11.237	73.556	4
P0DJI9	Serum amyloid A-2 protein OS=Homo sapiens GN=SAA2	SAA2	13.527	15.484	2
P10124	Serglycin OS=Homo sapiens GN=SRGN	SRGN	17.652	18.851	1
P10253	Lysosomal alpha-glucosidase OS=Homo sapiens GN=GAA	GAA	105.32	6.5313	1
P10451	Osteopontin OS=Homo sapiens GN=SPP1	SPP1	35.422	39.036	1
P10586	Receptor-type tyrosine-protein phosphatase F OS=Homo sapiens GN=PTPRF	PTPRF	212.88	78.661	9
P10599	Thioredoxin OS=Homo sapiens GN=TXN	TXN	11.737	43.665	4
P10619	Lysosomal protective protein OS=Homo sapiens GN=CTSA	CTSA	54.465	11.32	1
P10643	Complement component C7 OS=Homo sapiens GN=C7	C7	93.517	323.31	23
P10645	Chromogranin-A OS=Homo sapiens GN=CHGA	CHGA	50.688	18.506	2
P10646	Tissue factor pathway inhibitor OS=Homo sapiens GN=TFPI	TFPI	35.015	77.351	4
P10720	Platelet factor 4 variant OS=Homo sapiens GN=PF4V1	PF4V1	11.553	58.231	3
P10721	Mast/stem cell growth factor receptor Kit OS=Homo sapiens GN=KIT	KIT	109.86	35.664	4
P10768	S-formylglutathione hydrolase OS=Homo sapiens GN=ESD	ESD	31.462	12.695	2
P10909	Clusterin OS=Homo sapiens GN=CLU	CLU	52.494	312.15	11
P10912	Growth hormone receptor OS=Homo sapiens GN=GHR	GHR	71.499	6.2131	1
P11021	78 kDa glucose-regulated protein OS=Homo sapiens GN=HSPA5	HSPA5	72.332	158.44	14
P11047	Laminin subunit gamma-1 OS=Homo sapiens GN=LAMC1	LAMC1	177.6	34.045	5
P11142	Heat shock cognate 71 kDa protein OS=Homo sapiens GN=HSPA8	HSPA8	70.897	76.868	9
P11150	Hepatic triacylglycerol lipase OS=Homo sapiens GN=LIPC	LIPC	55.914	20.552	1
P11166	Solute carrier family 2, facilitated glucose transporter member 1 OS=Homo sapiens GN=SLC2A1	SLC2A1	54.083	7.5988	1
P11226	Mannose-binding protein C OS=Homo sapiens GN=MBL2	MBL2	26.143	131.76	5
P11277	Spectrin beta chain, erythrocytic OS=Homo sapiens GN=SPTB	SPTB	246.47	52.081	7
P11279	Lysosome-associated membrane glycoprotein 1 OS=Homo sapiens GN=LAMP1	LAMP1	44.882	36.589	3

P11488	Guanine nucleotide-binding protein G(t) subunit alpha-1 OS=Homo sapiens GN=GNAT1	GNAT1	40.04	6.8539	1
P11597	Cholesteryl ester transfer protein OS=Homo sapiens GN=CETP	CETP	54.756	35.281	4
P11717	Cation-independent mannose-6-phosphate receptor OS=Homo sapiens GN=IGF2R	IGF2R	274.37	95.419	13
P12107	Collagen alpha-1(XI) chain OS=Homo sapiens GN=COL11A1	COL11A1	181.06	7.2489	1
P12109	Collagen alpha-1(VI) chain OS=Homo sapiens GN=COL6A1	COL6A1	108.53	84.182	6
P12111	Collagen alpha-3(VI) chain OS=Homo sapiens GN=COL6A3	COL6A3	343.67	263.35	25
P12259	Coagulation factor V OS=Homo sapiens GN=F5	F5	251.7	323.31	44
P12273	Prolactin-inducible protein OS=Homo sapiens GN=PIP	PIP	16.572	15.436	2
P12814	Alpha-actinin-1 OS=Homo sapiens GN=ACTN1	ACTN1	103.06	11.837	1
P12821	Angiotensin-converting enzyme OS=Homo sapiens GN=ACE	ACE	149.71	46.706	7
P12830	Cadherin-1 OS=Homo sapiens GN=CDH1	CDH1	97.455	74.868	6
P12955	Xaa-Pro dipeptidase OS=Homo sapiens GN=PEPD	PEPD	54.548	47.403	6
P13473	Lysosome-associated membrane glycoprotein 2 OS=Homo sapiens GN=LAMP2	LAMP2	44.96	6.2206	1
P13489	Ribonuclease inhibitor OS=Homo sapiens GN=RNH1	RNH1	49.973	21.575	3
P13497	Bone morphogenetic protein 1 OS=Homo sapiens GN=BMP1	BMP1	111.25	7.7392	1
P13591	Neural cell adhesion molecule 1 OS=Homo sapiens GN=NCAM1	NCAM1	94.573	211.27	17
P13598	Intercellular adhesion molecule 2 OS=Homo sapiens GN=ICAM2	ICAM2	30.654	7.3268	1
P13611	Versican core protein OS=Homo sapiens GN=VCAN	VCAN	372.82	19.393	3
P13637	Sodium/potassium-transporting ATPase subunit alpha-3 OS=Homo sapiens GN=ATP1A3	ATP1A3	111.75	55.214	2
P13671	Complement component C6 OS=Homo sapiens GN=C6	C6	104.79	323.31	31
P13716	Delta-aminolevulinic acid dehydratase OS=Homo sapiens GN=ALAD	ALAD	36.294	121.32	6
P13727	Bone marrow proteoglycan OS=Homo sapiens GN=PRG2	PRG2	25.205	12.673	2
P13746	HLA class I histocompatibility antigen, A-11 alpha chain OS=Homo sapiens GN=HLA-A	HLA-A	40.936	23.086	3
P13796	Plastin-2 OS=Homo sapiens GN=LCP1	LCP1	70.288	183.01	16
P13797	Plastin-3 OS=Homo sapiens GN=PLS3	PLS3	70.81	-2	4
P13942	Collagen alpha-2(XI) chain OS=Homo sapiens GN=COL11A2	COL11A2	171.79	28.904	2
P13987	CD59 glycoprotein OS=Homo sapiens GN=CD59	CD59	14.177	20.134	2
P14151	L-selectin OS=Homo sapiens GN=SELL	SELL	42.187	188.06	7
P14174	Macrophage migration inhibitory factor OS=Homo sapiens GN=MIF	MIF	12.476	7.0299	1
P14314	Glucosidase 2 subunit beta OS=Homo sapiens GN=PRKCSH	PRKCSH	59.425	36.022	4
P14543	Nidogen-1 OS=Homo sapiens GN=NID1	NID1	136.38	106.99	11
P14618	Pyruvate kinase PKM OS=Homo sapiens GN=PKM	PKM	57.936	100.66	10
P14625	Endoplasmin OS=Homo sapiens GN=HSP90B1	HSP90B1	92.468	146.09	12
P14780	Matrix metalloproteinase-9 OS=Homo sapiens GN=MMP9	MMP9	78.457	63.123	8
P14923	Junction plakoglobin OS=Homo sapiens GN=JUP	JUP	81.744	122.73	13
P15088	Mast cell carboxypeptidase A OS=Homo sapiens GN=CPA3	CPA3	48.669	6.3017	1
P15144	Aminopeptidase N OS=Homo sapiens GN=ANPEP	ANPEP	109.54	150.74	14
P15151	Poliovirus receptor OS=Homo sapiens GN=PVR	PVR	45.302	19.95	2
P15169	Carboxypeptidase N catalytic chain OS=Homo sapiens GN=CPN1	CPN1	52.286	129.19	8
P15924	Desmoplakin OS=Homo sapiens GN=DSP	DSP	331.77	285.62	35
P16035	Metalloproteinase inhibitor 2 OS=Homo sapiens GN=TIMP2	TIMP2	24.399	71.867	4
P16070	CD44 antigen OS=Homo sapiens GN=CD44	CD44	81.537	12.122	2
P16109	P-selectin OS=Homo sapiens GN=SELP	SELP	90.833	34.015	3
P16112	Aggrecan core protein OS=Homo sapiens GN=ACAN	ACAN	250.19	59.468	5
P16157	Ankyrin-1 OS=Homo sapiens GN=ANK1	ANK1	206.26	76.457	9
P16284	Platelet endothelial cell adhesion molecule OS=Homo sapiens GN=PECAM1	PECAM1	82.535	12.022	2
P16403	Histone H1.2 OS=Homo sapiens GN=HIST1H1C	HIST1H1C	21.364	6.6777	1
P17066	Heat shock 70 kDa protein 6 OS=Homo sapiens GN=HSPA6	HSPA6	71.027	27.665	4
P17174	Aspartate aminotransferase, cytoplasmic OS=Homo sapiens GN=GOT1	GOT1	46.247	18.482	1
P17301	Integrin alpha-2 OS=Homo sapiens GN=ITGA2	ITGA2	129.29	56.65	7
P17600	Synapsin-1 OS=Homo sapiens GN=SYN1	SYN1	74.111	22.228	1
P17813	Endoglin OS=Homo sapiens GN=ENG	ENG	70.577	48.622	6
P17858	ATP-dependent 6-phosphofructokinase, liver type OS=Homo sapiens GN=PFKL	PFKL	85.018	21.928	2
P17931	Galectin-3 OS=Homo sapiens GN=LGALS3	LGALS3	26.152	18.405	2
P17936	Insulin-like growth factor-binding protein 3 OS=Homo sapiens GN=IGFBP3	IGFBP3	31.674	295.36	14
P17980	26S protease regulatory subunit 6A OS=Homo sapiens GN=PSMC3	PSMC3	49.203	6.4578	1
P17987	T-complex protein 1 subunit alpha OS=Homo sapiens GN=TCP1	TCP1	60.343	17.852	2
P18065	Insulin-like growth factor-binding protein 2 OS=Homo sapiens GN=IGFBP2	IGFBP2	34.814	60.297	5
P18084	Integrin beta-5 OS=Homo sapiens GN=ITGB5	ITGB5	88.053	6.4687	1
P18206	Vinculin OS=Homo sapiens GN=VCL	VCL	123.8	183.9	21
P18428	Lipopolysaccharide-binding protein OS=Homo sapiens GN=LBP	LBP	53.383	41.685	4
P18564	Integrin beta-6 OS=Homo sapiens GN=ITGB6	ITGB6	85.935	6.3256	1
P18850	Cyclic AMP-dependent transcription factor ATF-6 alpha OS=Homo sapiens GN=ATF6	ATF6	74.584	19.23	3
P19021	Peptidyl-glycine alpha-amidating monooxygenase OS=Homo sapiens GN=PAM	PAM	108.33	12.07	2
P19022	Cadherin-2 OS=Homo sapiens GN=CDH2	CDH2	99.808	34.446	5
P19256	Lymphocyte function-associated antigen 3 OS=Homo sapiens GN=CD58	CD58	28.147	8.1356	1
P19320	Vascular cell adhesion protein 1 OS=Homo sapiens GN=VCAM1	VCAM1	81.275	131.92	15
P19367	Hexokinase-1 OS=Homo sapiens GN=HK1	HK1	102.48	10.804	1
P19652	Alpha-1-acid glycoprotein 2 OS=Homo sapiens GN=ORM2	ORM2	23.602	102.98	6
P19823	Inter-alpha-trypsin inhibitor heavy chain H2 OS=Homo sapiens GN=ITIH2	ITIH2	106.46	323.31	25
P19827	Inter-alpha-trypsin inhibitor heavy chain H1 OS=Homo sapiens GN=ITIH1	ITIH1	101.39	323.31	17
P19961	Alpha-amylase 2B OS=Homo sapiens GN=AMY2B	AMY2B	57.709	111.85	6
P19971	Thymidine phosphorylase OS=Homo sapiens GN=TYMP	TYMP	49.955	7.2286	1
P20023	Complement receptor type 2 OS=Homo sapiens GN=CR2	CR2	112.91	85.399	10
P20742	Pregnancy zone protein OS=Homo sapiens GN=PZP	PZP	163.86	149.06	22
P20774	Mimecan OS=Homo sapiens GN=OGN	OGN	33.922	11.835	2
P20810	Calpastatin OS=Homo sapiens GN=CAST	CAST	76.572	8.2661	1
P20851	C4b-binding protein beta chain OS=Homo sapiens GN=C4BPB	C4BPB	28.357	68.652	8
P21291	Cysteine and glycine-rich protein 1 OS=Homo sapiens GN=CSRP1	CSRP1	20.567	9.3979	1
P21695	Glycerol-3-phosphate dehydrogenase [NAD(+)], cytoplasmic OS=Homo sapiens GN=GPD1	GPD1	37.567	7.1783	1
P21796	Voltage-dependent anion-selective channel protein 1 OS=Homo sapiens GN=VDAC1	VDAC1	30.772	13.309	1
P22105	Tenascin-X OS=Homo sapiens GN=TNXB	TNXB	458.22	323.31	32
P22304	Iduronate 2-sulfatase OS=Homo sapiens GN=IDS	IDS	61.872	6.4037	1
P22314	Ubiquitin-like modifier-activating enzyme 1 OS=Homo sapiens GN=UBA1	UBA1	117.85	34.417	4
P22352	Glutathione peroxidase 3 OS=Homo sapiens GN=GPX3	GPX3	25.552	148.37	9
P22392	Nucleoside diphosphate kinase B OS=Homo sapiens GN=NME2	NME2	17.298	30.898	4
P22692	Insulin-like growth factor-binding protein 4 OS=Homo sapiens GN=IGFBP4	IGFBP4	27.934	21.673	3
P22792	Carboxypeptidase N subunit 2 OS=Homo sapiens GN=CPN2	CPN2	60.556	239.2	9
P22891	Vitamin K-dependent protein Z OS=Homo sapiens GN=PROZ	PROZ	44.743	52.311	6

P22897	Macrophage mannose receptor 1 OS=Homo sapiens GN=MRC1	MRC1	166.01	58.86	8
P23142	Fibulin-1 OS=Homo sapiens GN=FBLN1	FBLN1	77.213	205.47	11
P23284	Peptidyl-prolyl cis-trans isomerase B OS=Homo sapiens GN=PPIB	PPIB	23.742	28.478	4
P23381	Tryptophan--tRNA ligase, cytoplasmic OS=Homo sapiens GN=WARS	WARS	53.165	25.919	3
P23467	Receptor-type tyrosine-protein phosphatase beta OS=Homo sapiens GN=PTPRB	PTPRB	224.3	6.8328	1
P23470	Receptor-type tyrosine-protein phosphatase gamma OS=Homo sapiens GN=PTPRG	PTPRG	162	52.82	7
P23471	Receptor-type tyrosine-protein phosphatase zeta OS=Homo sapiens GN=PTPRZ1	PTPRZ1	254.58	35.56	5
P23490	Loricrin OS=Homo sapiens GN=LOR	LOR	25.76	7.1	1
P23526	Adenosylhomocysteinase OS=Homo sapiens GN=AHCY	AHCY	47.716	20.362	3
P23528	Cofilin-1 OS=Homo sapiens GN=CFL1	CFL1	18.502	36.344	4
P24043	Laminin subunit alpha-2 OS=Homo sapiens GN=LAMA2	LAMA2	343.9	46.871	5
P24298	Alanine aminotransferase 1 OS=Homo sapiens GN=GPT	GPT	54.636	6.3009	1
P24387	Corticotropin-releasing factor-binding protein OS=Homo sapiens GN=CRHBP	CRHBP	36.144	34.619	5
P24592	Insulin-like growth factor-binding protein 6 OS=Homo sapiens GN=IGFBP6	IGFBP6	25.322	81.45	4
P24593	Insulin-like growth factor-binding protein 5 OS=Homo sapiens GN=IGFBP5	IGFBP5	30.57	76.481	8
P24666	Low molecular weight phosphotyrosine protein phosphatase OS=Homo sapiens GN=ACP1	ACP1	18.042	31.555	2
P24821	Tenascin OS=Homo sapiens GN=TNC	TNC	240.85	268.34	18
P25311	Zinc-alpha-2-glycoprotein OS=Homo sapiens GN=AZGP1	AZGP1	34.258	323.31	17
P25325	3-mercaptopyruvate sulfurtransferase OS=Homo sapiens GN=MPST	MPST	33.178	6.2794	1
P26038	Moesin OS=Homo sapiens GN=MSN	MSN	67.819	56.482	9
P26447	Protein S100-A4 OS=Homo sapiens GN=S100A4	S100A4	11.728	18.68	3
P26927	Hepatocyte growth factor-like protein OS=Homo sapiens GN=MST1	MST1	80.319	323.31	15
P26992	Ciliary neurotrophic factor receptor subunit alpha OS=Homo sapiens GN=CNTFR	CNTFR	40.633	16.791	2
P27169	Serum paraoxonase/arylesterase 1 OS=Homo sapiens GN=PON1	PON1	39.731	142.84	6
P27487	Dipeptidyl peptidase 4 OS=Homo sapiens GN=DPP4	DPP4	88.278	18.569	3
P27797	Calreticulin OS=Homo sapiens GN=CALR	CALR	48.141	56.55	6
P27918	Properdin OS=Homo sapiens GN=CFP	CFP	51.276	182.29	15
P27930	Interleukin-1 receptor type 2 OS=Homo sapiens GN=IL1R2	IL1R2	45.421	17.806	2
P28799	Granulins OS=Homo sapiens GN=GRN	GRN	63.544	42.392	6
P28827	Receptor-type tyrosine-protein phosphatase mu OS=Homo sapiens GN=PTPRM	PTPRM	163.68	48.848	4
P29401	Transketolase OS=Homo sapiens GN=TKT	TKT	67.877	30.185	5
P29622	Kallistatin OS=Homo sapiens GN=SERPINA4	SERPINA4	48.541	253.31	14
P30041	Peroxiredoxin-6 OS=Homo sapiens GN=PRDX6	PRDX6	25.035	35.41	5
P30043	Flavin reductase (NADPH) OS=Homo sapiens GN=BLVRB	BLVRB	22.119	95.352	6
P30044	Peroxiredoxin-5, mitochondrial OS=Homo sapiens GN=PRDX5	PRDX5	22.086	11.852	2
P30046	D-dopachrome decarboxylase OS=Homo sapiens GN=DDT	DDT	12.712	12.482	2
P30086	Phosphatidylethanolamine-binding protein 1 OS=Homo sapiens GN=PEBP1	PEBP1	21.057	22.363	3
P30101	Protein disulfide-isomerase A3 OS=Homo sapiens GN=PDIA3	PDIA3	56.782	58.383	4
P30530	Tyrosine-protein kinase receptor UFO OS=Homo sapiens GN=AXL	AXL	98.335	11.339	1
P30566	Adenylosuccinate lyase OS=Homo sapiens GN=ADSL	ADSL	54.889	12.721	2
P31146	Coronin-1A OS=Homo sapiens GN=CORO1A	CORO1A	51.026	36.657	2
P31151	Protein S100-A7 OS=Homo sapiens GN=S100A7	S100A7	11.471	13.836	2
P31944	Caspase-14 OS=Homo sapiens GN=CASP14	CASP14	27.679	49.399	4
P31947	14-3-3 protein sigma OS=Homo sapiens GN=SFN	SFN	27.774	6.7972	2
P31948	Stress-induced-phosphoprotein 1 OS=Homo sapiens GN=STIP1	STIP1	62.639	11.576	2
P32004	Neural cell adhesion molecule L1 OS=Homo sapiens GN=L1CAM	L1CAM	140	57.621	4
P32119	Peroxiredoxin-2 OS=Homo sapiens GN=PRDX2	PRDX2	21.892	67.763	8
P32754	4-hydroxyphenylpyruvate dioxygenase OS=Homo sapiens GN=HPD	HPD	44.934	6.6935	1
P32942	Intercellular adhesion molecule 3 OS=Homo sapiens GN=ICAM3	ICAM3	59.54	7.2661	1
P33151	Cadherin-5 OS=Homo sapiens GN=CDH5	CDH5	87.527	106.59	9
P33908	Mannosyl-oligosaccharide 1,2-alpha-mannosidase IA OS=Homo sapiens GN=MAN1A1	MAN1A1	72.968	56.493	8
P34096	Ribonuclease 4 OS=Homo sapiens GN=RNASE4	RNASE4	16.84	18.581	1
P34932	Heat shock 70 kDa protein 4 OS=Homo sapiens GN=HSPA4	HSPA4	94.33	30.931	5
P35443	Thrombospondin-4 OS=Homo sapiens GN=THBS4	THBS4	105.87	323.31	14
P35542	Serum amyloid A-4 protein OS=Homo sapiens GN=SAA4	SAA4	14.746	26.606	4
P35555	Fibrillin-1 OS=Homo sapiens GN=FBN1	FBN1	312.24	291.48	30
P35579	Myosin-9 OS=Homo sapiens GN=MYH9	MYH9	226.53	13.297	2
P35590	Tyrosine-protein kinase receptor Tie-1 OS=Homo sapiens GN=TIE1	TIE1	125.09	41.999	4
P35754	Glutaredoxin-1 OS=Homo sapiens GN=GLRX	GLRX	11.776	6.9009	1
P35858	Insulin-like growth factor-binding protein complex acid labile subunit OS=Homo sapiens GN=IGFALS	IGFALS	66.034	121.36	14
P35916	Vascular endothelial growth factor receptor 3 OS=Homo sapiens GN=FLT4	FLT4	152.76	83.302	6
P36871	Phosphoglucomutase-1 OS=Homo sapiens GN=PGM1	PGM1	61.448	23.964	4
P36955	Pigment epithelium-derived factor OS=Homo sapiens GN=SERPINF1	SERPINF1	46.312	212.95	12
P36980	Complement factor H-related protein 2 OS=Homo sapiens GN=CFHR2	CFHR2	30.65	129.63	10
P37802	Transgelin-2 OS=Homo sapiens GN=TAGLN2	TAGLN2	22.391	12.257	2
P37837	Transaldolase OS=Homo sapiens GN=TALDO1	TALDO1	37.54	28.981	3
P37840	Alpha-synuclein OS=Homo sapiens GN=SNCA	SNCA	14.46	63.26	3
P39059	Collagen alpha-1(XV) chain OS=Homo sapiens GN=COL15A1	COL15A1	141.72	8.6699	1
P39060	Collagen alpha-1(XVIII) chain OS=Homo sapiens GN=COL18A1	COL18A1	178.19	22.693	2
P40189	Interleukin-6 receptor subunit beta OS=Homo sapiens GN=IL6ST	IL6ST	103.54	25.695	4
P40197	Platelet glycoprotein V OS=Homo sapiens GN=GP5	GP5	60.958	6.8016	1
P40925	Malate dehydrogenase, cytoplasmic OS=Homo sapiens GN=MDH1	MDH1	36.426	91.997	8
P40926	Malate dehydrogenase, mitochondrial OS=Homo sapiens GN=MDH2	MDH2	35.503	82.558	2
P41222	Prostaglandin-H2 D-isomerase OS=Homo sapiens GN=PTGDS	PTGDS	21.029	11.603	2
P42785	Lysosomal Pro-X carboxypeptidase OS=Homo sapiens GN=PRCP	PRCP	55.799	43.136	5
P43121	Cell surface glycoprotein MUC18 OS=Homo sapiens GN=MCAM	MCAM	71.607	78.918	5
P43251	Biotinidase OS=Homo sapiens GN=BTD	BTD	61.132	99.55	6
P43304	Glycerol-3-phosphate dehydrogenase, mitochondrial OS=Homo sapiens GN=GPD2	GPD2	80.852	6.336	1
P43652	Afamin OS=Homo sapiens GN=AFM	AFM	69.068	323.31	28
P45880	Voltage-dependent anion-selective channel protein 2 OS=Homo sapiens GN=VDAC2	VDAC2	31.566	125.79	1
P46531	Neurogenic locus notch homolog protein 1 OS=Homo sapiens GN=NOTCH1	NOTCH1	272.5	18.966	3
P46926	Glucosamine-6-phosphate isomerase 1 OS=Homo sapiens GN=GNPDA1	GNPDA1	32.668	6.268	1
P47756	F-actin-capping protein subunit beta OS=Homo sapiens GN=CAPZB	CAPZB	31.35	8.9129	1
P48506	Glutamate--cysteine ligase catalytic subunit OS=Homo sapiens GN=GCLC	GCLC	72.765	35.536	3
P48637	Glutathione synthetase OS=Homo sapiens GN=GSS	GSS	52.384	53.22	4
P48740	Mannan-binding lectin serine protease 1 OS=Homo sapiens GN=MASP1	MASP1	79.246	242.91	14
P48745	Protein NOV homolog OS=Homo sapiens GN=NOV	NOV	39.162	13.06	2
P49257	Protein ERGIC-53 OS=Homo sapiens GN=LMAN1	LMAN1	57.548	8.0975	1
P49368	T-complex protein 1 subunit gamma OS=Homo sapiens GN=CCT3	CCT3	60.533	6.4237	1

P49641	Alpha-mannosidase 2x OS=Homo sapiens GN=MAN2A2	MAN2A2	130.54	25.141	4
P49747	Cartilage oligomeric matrix protein OS=Homo sapiens GN=COMP	COMP	82.86	323.31	14
P49908	Selenoprotein P OS=Homo sapiens GN=SEPP1	SEPP1	43.173	101.2	5
P49913	Cathelicidin antimicrobial peptide OS=Homo sapiens GN=CAMP	CAMP	19.301	44.329	5
P50395	Rab GDP dissociation inhibitor beta OS=Homo sapiens GN=GDI2	GDI2	50.663	55.706	6
P50990	T-complex protein 1 subunit theta OS=Homo sapiens GN=CCT8	CCT8	59.62	12.341	1
P50993	Sodium/potassium-transporting ATPase subunit alpha-2 OS=Homo sapiens GN=ATP1A2	ATP1A2	112.26	9.1007	2
P51693	Amyloid-like protein 1 OS=Homo sapiens GN=APLP1	APLP1	72.176	8.2877	1
P51884	Lumican OS=Homo sapiens GN=LUM	LUM	38.429	148.63	9
P52209	6-phosphogluconate dehydrogenase, decarboxylating OS=Homo sapiens GN=PGD	PGD	53.139	36.558	4
P52907	F-actin-capping protein subunit alpha-1 OS=Homo sapiens GN=CAPZA1	CAPZA1	32.922	8.4268	1
P53396	ATP-citrate synthase OS=Homo sapiens GN=ACLY	ACLY	120.84	11.397	2
P54108	Cysteine-rich secretory protein 3 OS=Homo sapiens GN=CRISP3	CRISP3	27.63	40.637	4
P54289	Voltage-dependent calcium channel subunit alpha-2/delta-1 OS=Homo sapiens GN=CACNA2D1	CACNA2D1	124.57	92.469	11
P54578	Ubiquitin carboxyl-terminal hydrolase 14 OS=Homo sapiens GN=USP14	USP14	56.068	13.754	2
P54725	UV excision repair protein RAD23 homolog A OS=Homo sapiens GN=RAD23A	RAD23A	39.609	6.7066	1
P54764	Ephrin type-A receptor 4 OS=Homo sapiens GN=EPHA4	EPHA4	109.86	27.081	2
P54802	Alpha-N-acetylglucosaminidase OS=Homo sapiens GN=NAGLU	NAGLU	82.265	79.279	4
P55056	Apolipoprotein C-IV OS=Homo sapiens GN=APOC4	APOC4	14.553	58.648	7
P55058	Phospholipid transfer protein OS=Homo sapiens GN=PLTP	PLTP	54.739	51.916	4
P55072	Transitional endoplasmic reticulum ATPase OS=Homo sapiens GN=VCP	VCP	89.321	31.091	5
P55103	Inhibin beta C chain OS=Homo sapiens GN=INHBC	INHBC	38.237	69.549	4
P55268	Laminin subunit beta-2 OS=Homo sapiens GN=LAMB2	LAMB2	195.98	6.6321	1
P55285	Cadherin-6 OS=Homo sapiens GN=CDH6	CDH6	88.308	22.427	3
P55287	Cadherin-11 OS=Homo sapiens GN=CDH11	CDH11	87.964	19.837	3
P55290	Cadherin-13 OS=Homo sapiens GN=CDH13	CDH13	78.286	121.45	11
P55327	Tumor protein D52 OS=Homo sapiens GN=TPD52	TPD52	24.327	22.939	1
P56199	Integrin alpha-1 OS=Homo sapiens GN=ITGA1	ITGA1	130.85	6.6687	1
P58335	Anthrax toxin receptor 2 OS=Homo sapiens GN=ANTXR2	ANTXR2	53.666	39.848	4
P58546	Myotrophin OS=Homo sapiens GN=MTPN	MTPN	12.895	12.812	2
P60174	Triosephosphate isomerase OS=Homo sapiens GN=TPI1	TPI1	30.791	83.382	6
P60201	Myelin proteolipid protein OS=Homo sapiens GN=PLP1	PLP1	30.077	83.324	4
P61020	Ras-related protein Rab-5B OS=Homo sapiens GN=RAB5B	RAB5B	23.707	9.5427	1
P61106	Ras-related protein Rab-14 OS=Homo sapiens GN=RAB14	RAB14	23.897	7.8398	1
P61626	Lysozyme C OS=Homo sapiens GN=LYZ	LYZ	16.537	31.683	5
P61764	Syntaxin-binding protein 1 OS=Homo sapiens GN=STXBP1	STXBP1	67.568	17.817	1
P61769	Beta-2-microglobulin OS=Homo sapiens GN=B2M	B2M	13.714	11.668	2
P61916	Epididymal secretory protein E1 OS=Homo sapiens GN=NPC2	NPC2	16.57	37.008	3
P61981	14-3-3 protein gamma OS=Homo sapiens GN=YWHAG	YWHAG	28.302	43.34	6
P62158	Calmodulin OS=Homo sapiens GN=CALM1	CALM1	16.837	72.245	5
P62258	14-3-3 protein epsilon OS=Homo sapiens GN=YWHAE	YWHAE	29.174	20.149	4
P62760	Visinin-like protein 1 OS=Homo sapiens GN=VSNL1	VSNL1	22.142	6.2445	1
P62826	GTP-binding nuclear protein Ran OS=Homo sapiens GN=RAN	RAN	24.423	13.215	1
P62834	Ras-related protein Rap-1A OS=Homo sapiens GN=RAP1A	RAP1A	20.987	6.8036	1
P62837	Ubiquitin-conjugating enzyme E2 D2 OS=Homo sapiens GN=UBE2D2	UBE2D2	16.735	6.3107	1
P62873	Guanine nucleotide-binding protein G(I)/G(S)/G(T) subunit beta-1 OS=Homo sapiens GN=GNB1	GNB1	37.377	6.7491	1
P62917	60S ribosomal protein L8 OS=Homo sapiens GN=RPL8	RPL8	28.024	7.1562	1
P62937	Peptidyl-prolyl cis-trans isomerase A OS=Homo sapiens GN=PPIA	PPIA	18.012	37.156	6
P62942	Peptidyl-prolyl cis-trans isomerase FKBP1A OS=Homo sapiens GN=FKBP1A	FKBP1A	11.951	21.319	1
P62987	Ubiquitin-60S ribosomal protein L40 OS=Homo sapiens GN=UBA52	UBA52	14.728	27.644	3
P63104	14-3-3 protein zeta/delta OS=Homo sapiens GN=YWHAZ	YWHAZ	27.745	46.877	5
P63208	S-phase kinase-associated protein 1 OS=Homo sapiens GN=SKP1	SKP1	18.658	18.981	3
P63261	Actin, cytoplasmic 2 OS=Homo sapiens GN=ACTG1	ACTG1	41.792	192.24	11
P68366	Tubulin alpha-4A chain OS=Homo sapiens GN=TUBA4A	TUBA4A	49.924	32.751	3
P68871	Hemoglobin subunit beta OS=Homo sapiens GN=HBB	HBB	15.998	311.84	10
P69892	Hemoglobin subunit gamma-2 OS=Homo sapiens GN=HBG2	HBG2	16.126	114.87	8
P78324	Tyrosine-protein phosphatase non-receptor type substrate 1 OS=Homo sapiens GN=SIRPA	SIRPA	54.966	14.729	2
P78417	Glutathione S-transferase omega-1 OS=Homo sapiens GN=GSTO1	GSTO1	27.566	38.247	6
P78509	Reelin OS=Homo sapiens GN=RELN	RELN	388.38	24.444	4
P80108	Phosphatidylinositol-glycan-specific phospholipase D OS=Homo sapiens GN=GPLD1	GPLD1	92.335	223.29	15
P80188	Neutrophil gelatinase-associated lipocalin OS=Homo sapiens GN=LCN2	LCN2	22.588	19.364	3
P80723	Brain acid soluble protein 1 OS=Homo sapiens GN=BASP1	BASP1	22.693	91.349	6
P81605	Dermcidin OS=Homo sapiens GN=DCD	DCD	11.284	49.202	4
P84085	ADP-ribosylation factor 5 OS=Homo sapiens GN=ARF5	ARF5	20.529	7.2427	1
P98160	Basement membrane-specific heparan sulfate proteoglycan core protein OS=Homo sapiens GN=HSPG2	HSPG2	468.83	234.75	26
P98198	Phospholipid-transporting ATPase ID OS=Homo sapiens GN=ATP8B2	ATP8B2	137.44	-2	1
Q00610	Clathrin heavy chain 1 OS=Homo sapiens GN=CLTC	CLTC	191.61	11.899	2
Q01459	Di-N-acetylchitobiase OS=Homo sapiens GN=CTBS	CTBS	43.759	29.268	3
Q01469	Fatty acid-binding protein, epidermal OS=Homo sapiens GN=FABP5	FABP5	15.164	12.062	2
Q01518	Adenylyl cyclase-associated protein 1 OS=Homo sapiens GN=CAP1	CAP1	51.901	6.6176	1
Q01973	Inactive tyrosine-protein kinase transmembrane receptor ROR1 OS=Homo sapiens GN=ROR1	ROR1	104.28	6.555	1
Q02413	Desmoglein-1 OS=Homo sapiens GN=DSG1	DSG1	113.75	133.05	8
Q02487	Desmocollin-2 OS=Homo sapiens GN=DSC2	DSC2	99.961	8.6043	1
Q02818	Nucleobindin-1 OS=Homo sapiens GN=NUCB1	NUCB1	53.879	8.3137	1
Q02978	Mitochondrial 2-oxoglutarate/malate carrier protein OS=Homo sapiens GN=SLC25A11	SLC25A11	34.061	17.805	1
Q02985	Complement factor H-related protein 3 OS=Homo sapiens GN=CFHR3	CFHR3	37.323	99.823	5
Q03154	Aminoacylase-1 OS=Homo sapiens GN=ACY1	ACY1	45.884	14.027	2
Q03167	Transforming growth factor beta receptor type 3 OS=Homo sapiens GN=TGFBR3	TGFBR3	93.498	22.248	3
Q03591	Complement factor H-related protein 1 OS=Homo sapiens GN=CFHR1	CFHR1	37.65	6.5619	10
Q04721	Neurogenic locus notch homolog protein 2 OS=Homo sapiens GN=NOTCH2	NOTCH2	265.4	34.294	3
Q04756	Hepatocyte growth factor activator OS=Homo sapiens GN=HGFAC	HGFAC	70.681	210.03	12
Q05707	Collagen alpha-1(XIV) chain OS=Homo sapiens GN=COL14A1	COL14A1	193.51	9.1007	1
Q05C16	Leucine-rich repeat-containing protein 63 OS=Homo sapiens GN=LRRC63	LRRC63	66.308	7.0031	1
Q06033	Inter-alpha-trypsin inhibitor heavy chain H3 OS=Homo sapiens GN=ITIH3	ITIH3	99.848	277.19	19
Q06323	Proteasome activator complex subunit 1 OS=Homo sapiens GN=PSME1	PSME1	28.723	6.4687	1
Q06828	Fibromodulin OS=Homo sapiens GN=FMOD	FMOD	43.178	11.906	1
Q06830	Peroxiredoxin-1 OS=Homo sapiens GN=PRDX1	PRDX1	22.11	32.657	6
Q07075	Glutamyl aminopeptidase OS=Homo sapiens GN=ENPEP	ENPEP	109.24	12.626	2
Q07507	Dermatopontin OS=Homo sapiens GN=DPT	DPT	24.005	11.244	1

Q07954	Prolow-density lipoprotein receptor-related protein 1 OS=Homo sapiens GN=LRP1	LRP1	504.6	323.31	35
Q08188	Protein-glutamine gamma-glutamyltransferase E OS=Homo sapiens GN=TGM3	TGM3	76.631	7.1411	1
Q08257	Quinone oxidoreductase OS=Homo sapiens GN=CRYZ	CRYZ	35.206	7.0872	1
Q08380	Galectin-3-binding protein OS=Homo sapiens GN=LGALS3BP	LGALS3BP	65.33	303.68	14
Q08554	Desmocollin-1 OS=Homo sapiens GN=DSC1	DSC1	99.986	47.376	5
Q08830	Fibrinogen-like protein 1 OS=Homo sapiens GN=FGL1	FGL1	36.379	108.73	7
Q10469	Alpha-1,6-mannosyl-glycoprotein 2-beta-N-acetylglucosaminyltransferase OS=Homo sapiens GN=MGAT2	MGAT2	51.55	6.3208	1
Q10471	Polypeptide N-acetylgalactosaminyltransferase 2 OS=Homo sapiens GN=GALNT2	GALNT2	64.732	12.228	2
Q10588	ADP-ribosyl cyclase/cyclic ADP-ribose hydrolase 2 OS=Homo sapiens GN=BST1	BST1	35.724	12.461	2
Q12805	EGF-containing fibulin-like extracellular matrix protein 1 OS=Homo sapiens GN=EFEMP1	EFEMP1	54.64	129.77	10
Q12841	Follistatin-related protein 1 OS=Homo sapiens GN=FSTL1	FSTL1	34.985	29.573	5
Q12860	Contactin-1 OS=Homo sapiens GN=CNTN1	CNTN1	113.32	120.66	15
Q12864	Cadherin-17 OS=Homo sapiens GN=CDH17	CDH17	92.218	7.1263	1
Q12866	Tyrosine-protein kinase Mer OS=Homo sapiens GN=MERTK	MERTK	110.25	6.7747	1
Q12884	Prolyl endopeptidase FAP OS=Homo sapiens GN=FAP	FAP	87.711	52.134	7
Q12907	Vesicular integral-membrane protein VIP36 OS=Homo sapiens GN=LMAN2	LMAN2	40.228	22.233	3
Q12913	Receptor-type tyrosine-protein phosphatase eta OS=Homo sapiens GN=PTPRJ	PTPRJ	145.94	87.457	4
Q13093	Platelet-activating factor acetylhydrolase OS=Homo sapiens GN=PLA2G7	PLA2G7	50.077	19.524	3
Q13103	Secreted phosphoprotein 24 OS=Homo sapiens GN=SPP2	SPP2	24.337	49.993	5
Q13201	Multimerin-1 OS=Homo sapiens GN=MMRN1	MMRN1	138.11	93.774	11
Q13228	Selenium-binding protein 1 OS=Homo sapiens GN=SELENBP1	SELENBP1	52.39	59.803	7
Q13332	Receptor-type tyrosine-protein phosphatase S OS=Homo sapiens GN=PTPRS	PTPRS	217.04	81.315	7
Q13361	Microfibrillar-associated protein 5 OS=Homo sapiens GN=MFAP5	MFAP5	19.611	6.5426	1
Q13404	Ubiquitin-conjugating enzyme E2 variant 1 OS=Homo sapiens GN=UBE2V1	UBE2V1	16.495	19.503	2
Q13421	Mesothelin OS=Homo sapiens GN=MSLN	MSLN	68.985	29.057	3
Q13449	Limbic system-associated membrane protein OS=Homo sapiens GN=LSAMP	LSAMP	37.393	21.826	3
Q13508	Ecto-ADP-ribosyltransferase 3 OS=Homo sapiens GN=ART3	ART3	43.923	81.905	2
Q13509	Tubulin beta-3 chain OS=Homo sapiens GN=TUBB3	TUBB3	50.432	12.796	1
Q13740	CD166 antigen OS=Homo sapiens GN=ALCAM	ALCAM	65.102	74.838	6
Q13790	Apolipoprotein F OS=Homo sapiens GN=APOF	APOF	35.399	49.854	3
Q13822	Ectonucleotide pyrophosphatase/phosphodiesterase family member 2 OS=Homo sapiens GN=ENPP2	ENPP2	98.993	59.171	8
Q13867	Bleomycin hydrolase OS=Homo sapiens GN=BLMH	BLMH	52.562	17.033	2
Q14118	Dystroglycan OS=Homo sapiens GN=DAG1	DAG1	97.44	44.447	4
Q14126	Desmoglein-2 OS=Homo sapiens GN=DSG2	DSG2	122.29	22.773	3
Q14314	Fibroleukin OS=Homo sapiens GN=FGL2	FGL2	50.228	11.778	2
Q14393	Growth arrest-specific protein 6 OS=Homo sapiens GN=GAS6	GAS6	79.676	17.482	2
Q14515	SPARC-like protein 1 OS=Homo sapiens GN=SPARCL1	SPARCL1	75.207	107.37	9
Q14517	Protocadherin Fat 1 OS=Homo sapiens GN=FAT1	FAT1	506.27	6.2987	1
Q14520	Hyaluronan-binding protein 2 OS=Homo sapiens GN=HABP2	HABP2	62.671	293.77	15
Q14574	Desmocollin-3 OS=Homo sapiens GN=DSC3	DSC3	99.968	18.785	3
Q14624	Inter-alpha-trypsin inhibitor heavy chain H4 OS=Homo sapiens GN=ITIH4	ITIH4	103.36	323.31	25
Q14766	Latent-transforming growth factor beta-binding protein 1 OS=Homo sapiens GN=LTBP1	LTBP1	186.79	89.057	8
Q14956	Transmembrane glycoprotein NMB OS=Homo sapiens GN=GPNMB	GPNMB	63.922	22.317	3
Q15063	Periostin OS=Homo sapiens GN=POSTN	POSTN	93.313	180.95	16
Q15113	Procollagen C-endopeptidase enhancer 1 OS=Homo sapiens GN=PCOLCE	PCOLCE	47.972	175.14	11
Q15155	Nodal modulator 1 OS=Homo sapiens GN=NOMO1	NOMO1	134.32	34.942	4
Q15166	Serum paraoxonase/lactonase 3 OS=Homo sapiens GN=PON3	PON3	39.607	38.858	5
Q15223	Nectin-1 OS=Homo sapiens GN=NECTIN1	NECTIN1	57.157	20.251	3
Q15485	Ficolin-2 OS=Homo sapiens GN=FCN2	FCN2	34.001	7.6558	1
Q15582	Transforming growth factor-beta-induced protein ig-h3 OS=Homo sapiens GN=TGFBI	TGFBI	74.68	176.59	15
Q15828	Cystatin-M OS=Homo sapiens GN=CST6	CST6	16.511	8.0577	1
Q15848	Adiponectin OS=Homo sapiens GN=ADIPOQ	ADIPOQ	26.413	7.0184	1
Q16270	Insulin-like growth factor-binding protein 7 OS=Homo sapiens GN=IGFBP7	IGFBP7	29.13	65.78	5
Q16610	Extracellular matrix protein 1 OS=Homo sapiens GN=ECM1	ECM1	60.673	323.31	19
Q16620	BDNF/NT-3 growth factors receptor OS=Homo sapiens GN=NTRK2	NTRK2	91.998	8.9915	1
Q16627	C-C motif chemokine 14 OS=Homo sapiens GN=CCL14	CCL14	10.678	11.913	2
Q16706	Alpha-mannosidase 2 OS=Homo sapiens GN=MAN2A1	MAN2A1	131.14	220.16	9
Q16775	Hydroxyacylglutathione hydrolase, mitochondrial OS=Homo sapiens GN=HAGH	HAGH	33.805	7.9216	1
Q16851	UTP--glucose-1-phosphate uridylyltransferase OS=Homo sapiens GN=UGP2	UGP2	56.94	17.399	3
Q16853	Membrane primary amine oxidase OS=Homo sapiens GN=AOC3	AOC3	84.621	63.459	6
Q16881	Thioredoxin reductase 1, cytoplasmic OS=Homo sapiens GN=TXNRD1	TXNRD1	70.905	-2	1
Q17RW2	Collagen alpha-1(XXIV) chain OS=Homo sapiens GN=COL24A1	COL24A1	175.49	6.25	1
Q24JP5	Transmembrane protein 132A OS=Homo sapiens GN=TMEM132A	TMEM132A	110.11	6.9807	1
Q4G0P3	Hydrocephalus-inducing protein homolog OS=Homo sapiens GN=HYDIN	HYDIN	575.89	11.339	2
Q4LDE5	Sushi, von Willebrand factor type A, EGF and pentraxin domain-containing protein 1 OS=Homo sapiens GN=SVEP	SVEP1	390.17	24.509	4
Q537H7	Spermatogenesis-associated protein 45 OS=Homo sapiens GN=SPATA45	SPATA45	11.356	-2	1
Q5T749	Keratinocyte proline-rich protein OS=Homo sapiens GN=KPRP	KPRP	64.135	80.878	7
Q5VTE0	Putative elongation factor 1-alpha-like 3 OS=Homo sapiens GN=EEF1A1P5	EEF1A1P5	50.184	12.968	2
Q5VVQ6	Ubiquitin thioesterase OTU1 OS=Homo sapiens GN=YOD1	YOD1	38.322	6.2686	1
Q6EMK4	Vasorin OS=Homo sapiens GN=VASN	VASN	71.712	40.956	4
Q6NTF9	Rhomboid domain-containing protein 2 OS=Homo sapiens GN=RHBDD2	RHBDD2	39.202	-2	1
Q6NXT2	Histone H3.3C OS=Homo sapiens GN=H3F3C	H3F3C	15.214	6.7933	1
Q6P179	Endoplasmic reticulum aminopeptidase 2 OS=Homo sapiens GN=ERAP2	ERAP2	110.46	49.562	6
Q6P387	Uncharacterized protein C16orf46 OS=Homo sapiens GN=C16orf46	C16orf46	43.417	6.1822	1
Q6Q788	Apolipoprotein A-V OS=Homo sapiens GN=APOA5	APOA5	41.212	11.132	2
Q6UVK1	Chondroitin sulfate proteoglycan 4 OS=Homo sapiens GN=CSPG4	CSPG4	250.53	24.228	4
Q6UWP8	Suprabasin OS=Homo sapiens GN=SBSN	SBSN	60.54	161.13	6
Q6UX71	Plexin domain-containing protein 2 OS=Homo sapiens GN=PLXDC2	PLXDC2	59.582	38.069	5
Q6UXB8	Peptidase inhibitor 16 OS=Homo sapiens GN=PI16	PI16	49.471	37.954	5
Q6UY14	ADAMTS-like protein 4 OS=Homo sapiens GN=ADAMTSL4	ADAMTSL4	116.54	48.004	4
Q6XQN6	Nicotinate phosphoribosyltransferase OS=Homo sapiens GN=NAPRT	NAPRT	57.578	13.863	2
Q6YHK3	CD109 antigen OS=Homo sapiens GN=CD109	CD109	161.69	60.757	8
Q6ZSC3	RNA-binding protein 43 OS=Homo sapiens GN=RBM43	RBM43	40.666	11.183	2
Q76LX8	A disintegrin and metalloproteinase with thrombospondin motifs 13 OS=Homo sapiens GN=ADAMTS13	ADAMTS13	153.6	154.38	14
Q7L1Q6	Basic leucine zipper and W2 domain-containing protein 1 OS=Homo sapiens GN=BZW1	BZW1	48.043	6.2147	1
Q7Z5N4	Protein sidekick-1 OS=Homo sapiens GN=SDK1	SDK1	242.11	12.605	2
Q7Z7G0	Target of Nesh-SH3 OS=Homo sapiens GN=ABI3BP	ABI3BP	118.64	145.31	5
Q7Z7M0	Multiple epidermal growth factor-like domains protein 8 OS=Homo sapiens GN=MEGF8	MEGF8	303.1	86.143	10
Q86TH1	ADAMTS-like protein 2 OS=Homo sapiens GN=ADAMTSL2	ADAMTSL2	104.62	49.124	5

Q86TY3	Uncharacterized protein C14orf37 OS=Homo sapiens GN=C14orf37	C14orf37	84.172	7.9969	1
Q86U17	Serpin A11 OS=Homo sapiens GN=SERPINA11	SERPINA11	46.989	45.966	6
Q86UD1	Out at first protein homolog OS=Homo sapiens GN=OAF	OAF	30.688	14.748	2
Q86UN3	Reticulon-4 receptor-like 2 OS=Homo sapiens GN=RTN4RL2	RTN4RL2	46.105	18.605	1
Q86VB7	Scavenger receptor cysteine-rich type 1 protein M130 OS=Homo sapiens GN=CD163	CD163	125.45	219.92	15
Q86VP6	Cullin-associated NEDD8-dissociated protein 1 OS=Homo sapiens GN=CAND1	CAND1	136.37	19.356	3
Q86X29	Lipolysis-stimulated lipoprotein receptor OS=Homo sapiens GN=LSR	LSR	71.438	6.2378	1
Q86X40	Leucine-rich repeat-containing protein 28 OS=Homo sapiens GN=LRRC28	LRRC28	41.912	-2	1
Q8IUL8	Cartilage intermediate layer protein 2 OS=Homo sapiens GN=CILP2	CILP2	126.29	88.185	7
Q8IWV2	Contactin-4 OS=Homo sapiens GN=CNTN4	CNTN4	113.45	17.927	3
Q8IXL6	Extracellular serine/threonine protein kinase FAM20C OS=Homo sapiens GN=FAM20C	FAM20C	66.234	34.64	3
Q8IZF2	Adhesion G protein-coupled receptor F5 OS=Homo sapiens GN=ADGRF5	ADGRF5	149.46	82.59	8
Q8IZF6	Adhesion G-protein coupled receptor G4 OS=Homo sapiens GN=ADGRG4	ADGRG4	333.36	6.2505	1
Q8N9N2	Activating signal cointegrator 1 complex subunit 1 OS=Homo sapiens GN=ASCC1	ASCC1	45.509	-2	1
Q8NBJ4	Golgi membrane protein 1 OS=Homo sapiens GN=GOLM1	GOLM1	45.333	33.555	4
Q8NBP7	Proprotein convertase subtilisin/kexin type 9 OS=Homo sapiens GN=PCSK9	PCSK9	74.285	134.03	9
Q8NCC3	Group XV phospholipase A2 OS=Homo sapiens GN=PLA2G15	PLA2G15	46.657	10.054	1
Q8NDA2	Hemicentin-2 OS=Homo sapiens GN=HMCN2	HMCN2	541.97	20.048	3
Q8NE65	Protein ZNF738 OS=Homo sapiens GN=ZNF738	ZNF738	15.884	6.4254	1
Q8NFI4	Putative protein FAM10A5 OS=Homo sapiens GN=ST13P5	ST13P5	41.377	23.331	3
Q8TDL5	BPI fold-containing family B member 1 OS=Homo sapiens GN=BPIFB1	BPIFB1	52.441	12.249	2
Q8TDY8	Immunoglobulin superfamily DCC subclass member 4 OS=Homo sapiens GN=IGDCC4	IGDCC4	134.21	7.5381	1
Q8TER0	Sushi, nidogen and EGF-like domain-containing protein 1 OS=Homo sapiens GN=SNED1	SNED1	152.2	19.778	2
Q8WUA8	Tsukushin OS=Homo sapiens GN=TSKU	TSKU	37.807	9.1884	1
Q8WUM4	Programmed cell death 6-interacting protein OS=Homo sapiens GN=PDCD6IP	PDCD6IP	96.022	12.601	2
Q8WWA0	Intelectin-1 OS=Homo sapiens GN=ITLN1	ITLN1	34.961	6.3594	1
Q8WWZ8	Oncoprotein-induced transcript 3 protein OS=Homo sapiens GN=OIT3	OIT3	60.021	40.421	5
Q8WZ75	Roundabout homolog 4 OS=Homo sapiens GN=ROBO4	ROBO4	107.46	38.46	1
Q92484	Acid sphingomyelinase-like phosphodiesterase 3a OS=Homo sapiens GN=SMPDL3A	SMPDL3A	51.26	19.36	3
Q92496	Complement factor H-related protein 4 OS=Homo sapiens GN=CFHR4	CFHR4	65.35	48.902	4
Q92520	Protein FAM3C OS=Homo sapiens GN=FAM3C	FAM3C	24.68	24.078	3
Q92752	Tenascin-R OS=Homo sapiens GN=TNR	TNR	149.56	-2	1
Q92823	Neuronal cell adhesion molecule OS=Homo sapiens GN=NRCAM	NRCAM	143.89	29.896	4
Q92859	Neogenin OS=Homo sapiens GN=NEO1	NEO1	160.01	46.651	6
Q92954	Proteoglycan 4 OS=Homo sapiens GN=PRG4	PRG4	151.08	121.69	17
Q93063	Exostosin-2 OS=Homo sapiens GN=EXT2	EXT2	82.254	27.354	4
Q969T9	WW domain-binding protein 2 OS=Homo sapiens GN=WBP2	WBP2	28.087	40.824	1
Q96BZ4	Phospholipase D4 OS=Homo sapiens GN=PLD4	PLD4	55.626	6.5669	1
Q96EE4	Coiled-coil domain-containing protein 126 OS=Homo sapiens GN=CCDC126	CCDC126	15.668	11.702	2
Q96IY4	Carboxypeptidase B2 OS=Homo sapiens GN=CPB2	CPB2	48.424	42.8	5
Q96KG7	Multiple epidermal growth factor-like domains protein 10 OS=Homo sapiens GN=MEGF10	MEGF10	122.2	27.972	3
Q96KN2	Beta-Ala-His dipeptidase OS=Homo sapiens GN=CNDP1	CNDP1	56.705	250.35	10
Q96NZ9	Proline-rich acidic protein 1 OS=Homo sapiens GN=PRAP1	PRAP1	17.207	6.5876	1
Q96PD5	N-acetylmuramoyl-L-alanine amidase OS=Homo sapiens GN=PGLYRP2	PGLYRP2	62.216	323.31	8
Q96S96	Phosphatidylethanolamine-binding protein 4 OS=Homo sapiens GN=PEBP4	PEBP4	25.733	22.508	3
Q99435	Protein kinase C-binding protein NELL2 OS=Homo sapiens GN=NELL2	NELL2	91.346	13.529	2
Q99497	Protein deglycase DJ-1 OS=Homo sapiens GN=PARK7	PARK7	19.891	25.818	4
Q99650	Oncostatin-M-specific receptor subunit beta OS=Homo sapiens GN=OSMR	OSMR	110.51	17.914	2
Q99715	Collagen alpha-1(XII) chain OS=Homo sapiens GN=COL12A1	COL12A1	333.14	60.782	9
Q99784	Noelin OS=Homo sapiens GN=OLFM1	OLFM1	55.342	73.618	6
Q99878	Histone H2A type 1-J OS=Homo sapiens GN=HIST1H2AJ	HIST1H2AJ	13.936	11.836	2
Q99880	Histone H2B type 1-L OS=Homo sapiens GN=HIST1H2BL	HIST1H2BL	13.952	7.7241	1
Q99969	Retinoic acid receptor responder protein 2 OS=Homo sapiens GN=RARRES2	RARRES2	18.617	11.647	1
Q99972	Myocilin OS=Homo sapiens GN=MYOC	MYOC	56.971	26.145	4
Q99983	Osteomodulin OS=Homo sapiens GN=OMD	OMD	49.492	33.408	3
Q9BQ51	Programmed cell death 1 ligand 2 OS=Homo sapiens GN=PDCD1LG2	PDCD1LG2	30.957	6.4107	1
Q9BRA2	Thioredoxin domain-containing protein 17 OS=Homo sapiens GN=TXNDC17	TXNDC17	13.941	7.0059	1
Q9BS26	Endoplasmic reticulum resident protein 44 OS=Homo sapiens GN=ERP44	ERP44	46.971	6.2146	1
Q9BTY2	Plasma alpha-L-fucosidase OS=Homo sapiens GN=FUCA2	FUCA2	54.066	37.411	6
Q9BUN1	Protein MENT OS=Homo sapiens GN=MENT	MENT	36.769	8.9064	1
Q9BWP8	Collectin-11 OS=Homo sapiens GN=COLEC11	COLEC11	28.665	82.279	4
Q9BXJ4	Complement C1q tumor necrosis factor-related protein 3 OS=Homo sapiens GN=C1QTNF3	C1QTNF3	26.994	10.011	1
Q9BXR6	Complement factor H-related protein 5 OS=Homo sapiens GN=CFHR5	CFHR5	64.419	148.84	12
Q9BY67	Cell adhesion molecule 1 OS=Homo sapiens GN=CADM1	CADM1	48.509	148.4	4
Q9BYE9	Cadherin-related family member 2 OS=Homo sapiens GN=CDHR2	CDHR2	141.54	14.326	2
Q9BYJ0	Fibroblast growth factor-binding protein 2 OS=Homo sapiens GN=FGFBP2	FGFBP2	24.581	27.347	4
Q9BYZ6	Rho-related BTB domain-containing protein 2 OS=Homo sapiens GN=RHOBTB2	RHOBTB2	82.625	6.3671	1
Q9H0E9	Bromodomain-containing protein 8 OS=Homo sapiens GN=BRD8	BRD8	135.33	-2	1
Q9H1U4	Multiple epidermal growth factor-like domains protein 9 OS=Homo sapiens GN=MEGF9	MEGF9	62.983	19.827	3
Q9H299	SH3 domain-binding glutamic acid-rich-like protein 3 OS=Homo sapiens GN=SH3BGRL3	SH3BGRL3	10.438	18.924	3
Q9H4A4	Aminopeptidase B OS=Homo sapiens GN=RNPEP	RNPEP	72.595	11.264	2
Q9H4A9	Dipeptidase 2 OS=Homo sapiens GN=DPEP2	DPEP2	53.305	48.687	4
Q9H4G4	Golgi-associated plant pathogenesis-related protein 1 OS=Homo sapiens GN=GLIPR2	GLIPR2	17.218	71.068	3
Q9H6X2	Anthrax toxin receptor 1 OS=Homo sapiens GN=ANTXR1	ANTXR1	62.788	6.6997	1
Q9H8J5	MANSC domain-containing protein 1 OS=Homo sapiens GN=MANSC1	MANSC1	46.81	8.9287	1
Q9HBB8	Cadherin-related family member 5 OS=Homo sapiens GN=CDHR5	CDHR5	88.222	71.516	3
Q9HBR0	Putative sodium-coupled neutral amino acid transporter 10 OS=Homo sapiens GN=SLC38A10	SLC38A10	119.76	117.3	7
Q9HBW9	Adhesion G protein-coupled receptor L4 OS=Homo sapiens GN=ADGRL4	ADGRL4	77.81	12.703	2
Q9HCB6	Spondin-1 OS=Homo sapiens GN=SPON1	SPON1	90.973	12.428	2
Q9HCD6	Protein TANC2 OS=Homo sapiens GN=TANC2	TANC2	219.65	-2	1
Q9HCL0	Protocadherin-18 OS=Homo sapiens GN=PCDH18	PCDH18	126.15	12.092	2
Q9HCU0	Endosialin OS=Homo sapiens GN=CD248	CD248	80.858	31.826	4
Q9HDC9	Adipocyte plasma membrane-associated protein OS=Homo sapiens GN=APMAP	APMAP	46.48	54.095	6
Q9NPG4	Protocadherin-12 OS=Homo sapiens GN=PCDH12	PCDH12	128.99	17.378	2
Q9NPH3	Interleukin-1 receptor accessory protein OS=Homo sapiens GN=IL1RAP	IL1RAP	65.418	49.313	6
Q9NPR2	Semaphorin-4B OS=Homo sapiens GN=SEMA4B	SEMA4B	92.192	52.36	4
Q9NPY3	Complement component C1q receptor OS=Homo sapiens GN=CD93	CD93	68.559	193.7	5
Q9NQ79	Cartilage acidic protein 1 OS=Homo sapiens GN=CRTAC1	CRTAC1	71.42	121.39	9
Q9NRW1	Ras-related protein Rab-6B OS=Homo sapiens GN=RAB6B	RAB6B	23.461	8.0338	1

Q9NS71	Gastrokine-1 OS=Homo sapiens GN=GKN1	GKN1	21.999	8.3274	1
Q9NT22	EMILIN-3 OS=Homo sapiens GN=EMILIN3	EMILIN3	82.646	7.1736	1
Q9NTK5	Obg-like ATPase 1 OS=Homo sapiens GN=OLA1	OLA1	44.743	12.369	2
Q9NTU7	Cerebellin-4 OS=Homo sapiens GN=CBLN4	CBLN4	21.808	34.747	2
Q9NX62	Inositol monophosphatase 3 OS=Homo sapiens GN=IMPAD1	IMPAD1	38.681	18.915	3
Q9NY15	Stabilin-1 OS=Homo sapiens GN=STAB1	STAB1	275.48	6.8618	1
Q9NY97	N-acetyllactosaminide beta-1,3-N-acetylglucosaminyltransferase 2 OS=Homo sapiens GN=B3GNT2	B3GNT2	46.021	30.536	2
Q9NZ08	Endoplasmic reticulum aminopeptidase 1 OS=Homo sapiens GN=ERAP1	ERAP1	107.23	11.421	2
Q9NZD4	Alpha-hemoglobin-stabilizing protein OS=Homo sapiens GN=AHSP	AHSP	11.84	22.907	3
Q9NZK5	Adenosine deaminase CECR1 OS=Homo sapiens GN=CECR1	CECR1	58.933	50.243	7
Q9NZP8	Complement C1r subcomponent-like protein OS=Homo sapiens GN=C1RL	C1RL	53.498	260.28	9
Q9P121	Neurotrimin OS=Homo sapiens GN=NTM	NTM	37.971	6.6173	1
Q9P232	Contactin-3 OS=Homo sapiens GN=CNTN3	CNTN3	112.88	6.1988	1
Q9UBP4	Dickkopf-related protein 3 OS=Homo sapiens GN=DKK3	DKK3	38.39	19.996	3
Q9UBQ6	Exostosin-like 2 OS=Homo sapiens GN=EXTL2	EXTL2	37.465	44.078	5
Q9UBR2	Cathepsin Z OS=Homo sapiens GN=CTSZ	CTSZ	33.868	39.558	3
Q9UBX1	Cathepsin F OS=Homo sapiens GN=CTSF	CTSF	53.365	13.356	2
Q9UBX5	Fibulin-5 OS=Homo sapiens GN=FBLN5	FBLN5	50.18	24.423	4
Q9UEU0	Vesicle transport through interaction with t-SNAREs homolog 1B OS=Homo sapiens GN=VTI1B	VTI1B	26.688	6.5075	1
Q9UEW3	Macrophage receptor MARCO OS=Homo sapiens GN=MARCO	MARCO	52.657	23.894	2
Q9UGM5	Fetuin-B OS=Homo sapiens GN=FETUB	FETUB	42.054	166.11	7
Q9UHG3	Prenylcysteine oxidase 1 OS=Homo sapiens GN=PCYOX1	PCYOX1	56.639	162.99	11
Q9UI42	Carboxypeptidase A4 OS=Homo sapiens GN=CPA4	CPA4	47.351	6.6004	1
Q9UJJ9	N-acetylglucosamine-1-phosphotransferase subunit gamma OS=Homo sapiens GN=GNPTG	GNPTG	33.973	65.938	3
Q9UK55	Protein Z-dependent protease inhibitor OS=Homo sapiens GN=SERPINA10	SERPINA10	50.706	62.929	7
Q9UKX5	Integrin alpha-11 OS=Homo sapiens GN=ITGA11	ITGA11	133.47	11.42	2
Q9ULI3	Protein HEG homolog 1 OS=Homo sapiens GN=HEG1	HEG1	147.46	20.008	3
Q9UM47	Neurogenic locus notch homolog protein 3 OS=Homo sapiens GN=NOTCH3	NOTCH3	243.63	39.507	4
Q9UNW1	Multiple inositol polyphosphate phosphatase 1 OS=Homo sapiens GN=MINPP1	MINPP1	55.051	105.39	6
Q9UPN9	E3 ubiquitin-protein ligase TRIM33 OS=Homo sapiens GN=TRIM33	TRIM33	122.53	6.6744	1
Q9Y240	C-type lectin domain family 11 member A OS=Homo sapiens GN=CLEC11A	CLEC11A	35.694	18.889	3
Q9Y3C8	Ubiquitin-fold modifier-conjugating enzyme 1 OS=Homo sapiens GN=UFC1	UFC1	19.458	6.9424	1
Q9Y490	Talin-1 OS=Homo sapiens GN=TLN1	TLN1	269.76	31.691	3
Q9Y4D7	Plexin-D1 OS=Homo sapiens GN=PLXND1	PLXND1	212	13.976	2
Q9Y4L1	Hypoxia up-regulated protein 1 OS=Homo sapiens GN=HYOU1	HYOU1	111.33	6.8712	1
Q9Y5C1	Angiopoietin-related protein 3 OS=Homo sapiens GN=ANGPTL3	ANGPTL3	53.637	46.475	5
Q9Y5Y7	Lymphatic vessel endothelial hyaluronic acid receptor 1 OS=Homo sapiens GN=LYVE1	LYVE1	35.213	49.105	5
Q9Y646	Carboxypeptidase Q OS=Homo sapiens GN=CPQ	CPQ	51.887	14.291	2
Q9Y689	ADP-ribosylation factor-like protein 5A OS=Homo sapiens GN=ARL5A	ARL5A	20.728	-2	1
Q9Y6D5	Brefeldin A-inhibited guanine nucleotide-exchange protein 2 OS=Homo sapiens GN=ARFGEF2	ARFGEF2	202.04	6.4129	1
Q9Y6R7	IgGFc-binding protein OS=Homo sapiens GN=FCGBP	FCGBP	572.01	76.262	9
Q9Y6Z7	Collectin-10 OS=Homo sapiens GN=COLEC10	COLEC10	30.705	85.177	4
