# Supplementary material for: TMT-Based Proteomic Analysis of Plasma from Children with Rolandic Epilepsy
Source: Dis Markers. 2020 Oct 7;2020:8840482. doi: 10.1155/2020/8840482 (PMC7563079; doi:10.1155/2020/8840482)
Supplement: Supplementary 4 — Table S3: the list of differentially expressed protein between the epilepsy and control groups by TMT-based proteomics analysis. [file 8840482.f4.pdf]

Table S3.The list of differentially expressed protein between epilepsy and control group by TMT-based proteomics analysis.

Protein	E1/C1	E2/C2	E3/C3	E4/C4	E5/C5	Down	Up	SUM	
P60201	Up			Down	Up	1	2	3	Up
P13489	Down			Down		2	0	2	Down
Q99972	Up		Up			0	2	2	Up
P10599	Down	Down	Down	Down	Up	4	1	5	Down
P00748	Up	Up	Up	Up		0	4	4	Up
P22105	Down			Down		2	0	2	Down
P01008	Down		Up			1	1	2	
P02751	Up	Down	Up		Up	1	3	4	Up
P12821	Down	Down	Up	Down		3	1	4	Down
P08123	Down	Down	Up			2	1	3	Down
P06276	Up			Down	Down	2	1	3	Down
P16157	Down	Down			Up	2	1	3	Down
P07942	Down			Down		2	0	2	Down
P01023	Down		Up	Down	Up	2	2	4	
P00441	Down	Down	Down	Down		4	0	4	Down
P17936	Down		Down	Up		2	1	3	Down
P48506	Down		Down	Down	Up	3	1	4	Down
P29622	Down	Down		Up		2	1	3	Down
P02452	Down	Down	Up			2	1	3	Down
Q12884	Down			Down		2	0	2	Down
P62158	Down	Down	Down	Down	Up	4	1	5	Down
Q06830	Down	Down	Down	Down	Up	4	1	5	Down
P32004	Down		Up		Down	2	1	3	Down
P06727	Down			Down	Down	3	0	3	Down
P02042	Down	Down	Down	Down	Up	4	1	5	Down
O43505	Down				Up	1	1	2	
P01024	Up		Down			1	1	2	
P61626	Up			Up		0	2	2	Up
P40925	Down	Down	Down	Down		4	0	4	Down
Q9NPH3	Down		Down			2	0	2	Down
P23142	Down	Down	Up			2	1	3	Down
P08195	Down		Up			1	1	2	
P13598	Up				Up	0	2	2	Up
P16035	Down	Down				2	0	2	Down
P02549	Down	Down		Down	Up	3	1	4	Down
P12111	Down	Down	Up			2	1	3	Down
P04004	Up				Down	1	1	2	
O95479	Down	Up				1	1	2	
P31151	Down	Up			Up	1	2	3	Up
P04075	Down	Down	Down	Down		4	0	4	Down
P49913	Up			Up	Down	1	2	3	Up
P02743	Up		Down	Up		1	2	3	Up
P60174	Down	Down			Up	2	1	3	Down
P02458	Down				Down	2	0	2	Down
Q08554	Down				Up	1	1	2	
P62987	Down	Down	Down	Down		4	0	4	Down
P02654	Up		Up	Down		1	2	3	Up
P00568	Down	Down	Down	Down		4	0	4	Down
O43866	Down		Up	Up		1	2	3	Up
Q04756	Up	Down		Down		2	1	3	Down
Q13421	Down		Up			1	1	2	
P22891	Up	Down				1	1	2	
Q96KG7	Down			Down		2	0	2	Down
P18428	Up	Down	Down	Up		2	2	4	
P08519	Down	Down		Down	Down	4	0	4	Down
P26447	Down	Down	Down	Down		4	0	4	Down
P07237	Down		Down		Up	2	1	3	Down
P01009	Down	Up	Up	Up	Down	2	3	5	
P69892	Down	Down		Down	Up	3	1	4	Down
P08253	Down		Up			1	1	2	
P54108	Up		Up	Up		0	3	3	Up
Q14766	Down			Up	Down	2	1	3	Down
P18850	Down		Up			1	1	2	
P23526	Down	Down	Down	Down	Up	4	1	5	Down
P14923	Down				Up	1	1	2	
P15924	Down				Up	1	1	2	
Q99983	Down			Down		2	0	2	Down
Q6UXB8	Down				Up	1	1	2	
P54289	Down		Up		Down	2	1	3	Down
P22392	Down	Down	Down	Down		4	0	4	Down
Q9HDC9	Up		Up	Down		1	2	3	Up
Q06033	Down				Up	1	1	2	
P00918	Down	Down	Down	Down	Up	4	1	5	Down
P07360	Up			Up		0	2	2	Up
Q01469	Down				Up	1	1	2	
P0DJI9	Up	Down	Down		Up	2	2	4	
P23471	Down			Down		2	0	2	Down
P07358	Up			Up		0	2	2	Up
P32119	Down	Down	Down	Down	Up	4	1	5	Down
P02775	Down	Up	Down	Up	Down	3	2	5	
P30041	Down	Down	Down	Down	Down	5	0	5	Down
Q13228	Down	Down		Down		3	0	3	Down
P63104	Down	Down			Up	2	1	3	Down
P02649	Up	Down				1	1	2	
Q96KN2	Up	Down				1	1	2	
Q6P179	Down	Up		Up	Down	2	2	4	
P04040	Down	Down	Down	Down		4	0	4	Down
Q9Y5Y7	Down		Down			2	0	2	Down
P01871	Down		Up	Up		1	2	3	Up
P07339	Up			Up		0	2	2	Up
P43652	Up			Up	Down	1	2	3	Up
P01780	Down			Up		1	1	2	
P08603	Up	Up	Up	Up		0	4	4	Up
P00558	Down	Down		Down		3	0	3	Down
P04278	Down		Up	Down	Up	2	2	4	
P02786	Down		Down			2	0	2	Down
O00391	Down				Down	2	0	2	Down
Q8NFI4	Down	Down		Down		3	0	3	Down
Q5T749	Down		Up		Up	1	2	3	Up
Q9BYZ6	Down		Down	Down		3	0	3	Down
O14791	Up			Up	Down	1	2	3	Up
P07359	Down	Up		Up		1	2	3	Up
P02750	Down	Up	Down	Up		2	2	4	

P01834	Down			Up	Down	2	1	3	Down
O15335	Down		Up			1	1	2	
Q08380	Up		Down		Down	2	1	3	Down
P05090	Down	Up		Down		2	1	3	Down
O15394	Down				Up	1	1	2	
Q13103	Down				Up	1	1	2	
P13591	Down		Up	Down		2	1	3	Down
Q13201	Down			Up	Down	2	1	3	Down
P0CG06	Down	Up			Down	2	1	3	Down
P01857	Down		Down		Down	3	0	3	Down
P05154	Down	Down				2	0	2	Down
B9A064	Down			Up	Down	2	1	3	Down
P68871	Down	Down		Down	Up	3	1	4	Down
P58546	Down			Down		2	0	2	Down
P31947	Down	Down		Down		3	0	3	Down
P30043	Down	Down		Down	Up	3	1	4	Down
P00738	Up	Down	Down	Up	Down	3	2	5	
P09960	Up	Down				1	1	2	
P11226	Down	Down	Up	Down	Down	4	1	5	Down
P02787	Down			Up		1	1	2	
P35858	Down			Up		1	1	2	
P43121	Up		Up	Down		1	2	3	Up
Q15063	Down		Up	Down	Down	3	1	4	Down
P07911	Down	Up	Up		Up	1	3	4	Up
P13716	Down	Down	Down	Down		4	0	4	Down
P13942	Down	Down	Down	Down		4	0	4	Down
Q02413	Down				Up	1	1	2	
Q03591	Up	Up			Up	0	3	3	Up
P24666	Down	Down				2	0	2	Down
Q6YHK3	Down			Down		2	0	2	Down
P14314	Down				Down	2	0	2	Down
P05156	Up	Up	Down		Up	1	3	4	Up
P02763	Up	Up	Down	Up	Up	1	4	5	Up
P07738	Down	Down		Down		3	0	3	Down
P04406	Down	Down	Down	Down	Up	4	1	5	Down
P01031	Up			Up		0	2	2	Up
P09486	Down	Up		Up	Down	2	2	4	
P19652	Up	Up	Down	Up		1	3	4	Up
P00915	Down	Down	Down	Down		4	0	4	Down
P14543	Down			Up		1	1	2	
Q6UY14	Down		Up	Down		2	1	3	Down
P50395	Down	Down	Down	Down		4	0	4	Down
P04196	Up		Down			1	1	2	
Q9Y6R7		Up		Down		1	1	2	
P02647		Down		Down		2	0	2	Down
P11142		Down	Down	Down		3	0	3	Down
P03950		Down		Up	Down	2	1	3	Down
P62258		Down		Down		2	0	2	Down
Q86UD1		Down		Up		1	1	2	
P14780		Down			Down	2	0	2	Down
P01011		Up			Up	0	2	2	Up
P12259		Down	Up		Down	2	1	3	Down
P36955		Down	Down			2	0	2	Down
Q6NXT2		Up	Down	Down	Up	2	2	4	
P02748		Up		Up		0	2	2	Up
P29401		Down	Down	Down		3	0	3	Down
P30566		Down		Down		2	0	2	Down
Q9BTY2		Down			Down	2	0	2	Down
P18065		Up	Up		Down	1	2	3	Up
Q15166		Down		Down	Up	2	1	3	Down
P05186		Up	Up			0	2	2	Up
P55072		Down	Down			2	0	2	Down
Q99497		Down		Down		2	0	2	Down
P24593		Down		Up		1	1	2	
P49908		Down		Down		2	0	2	Down
P01860		Up		Down	Down	2	1	3	Down
P09172		Up	Up	Up	Up	0	4	4	Up
Q92496		Up	Down			1	1	2	
Q4G0P3		Down	Down			2	0	2	Down
Q8NBP7		Down		Down		2	0	2	Down
P62937		Down	Down	Down	Up	3	1	4	Down
P55103		Down		Up		1	1	2	
P0C0L5		Up			Up	0	2	2	Up
Q9UK55		Down	Down			2	0	2	Down
P35916		Up		Up		0	2	2	Up
P27169		Down			Up	1	1	2	
Q9NZ08		Down	Up			1	1	2	
P16070		Up	Up			0	2	2	Up
P78417		Down		Down		2	0	2	Down
P06733		Down	Down		Up	2	1	3	Down
P06702		Up	Down	Up		1	2	3	Up
Q15113		Up	Up			0	2	2	Up
P30086		Down		Down		2	0	2	Down
P52209		Down		Down	Up	2	1	3	Down
Q9Y5C1		Down	Down		Up	2	1	3	Down
P54802		Down		Down	Up	2	1	3	Down
P36980		Up	Down	Down	Up	2	2	4	
P00352		Down	Down	Down		3	0	3	Down
P11277		Down		Down	Up	2	1	3	Down
P04275		Down	Up	Down		2	1	3	Down
P06732		Up		Down		1	1	2	
P26038		Down		Down		2	0	2	Down
P02656			Up	Down	Down	2	1	3	Down
P02675			Up	Up	Up	0	3	3	Up
Q86U17			Up		Down	1	1	2	
Q99878			Down		Up	1	1	2	
P35443			Up		Down	1	1	2	
P10643			Up	Up		0	2	2	Up
Q6UVK1			Up	Down		1	1	2	
P20742			Up	Up		0	2	2	Up
P19961			Up	Up		0	2	2	Up
P02745			Down		Up	1	1	2	
P02671			Up	Up	Up	0	3	3	Up
P55290			Up	Down		1	1	2	
P11597			Down	Down		2	0	2	Down

P00740	Down	Up			1	1	2	
P17813	Up	Down			1	1	2	
P02679	Up	Up	Up		0	3	3	Up
P02766		Up	Down		1	1	2	
P08185		Down	Up		1	1	2	
O15031		Up	Down		1	1	2	
P14625		Down	Down		2	0	2	Down
P04433		Up	Down		1	1	2	
P20023		Up	Up		0	2	2	Up

group	total
Up	46
unknown	12
Down	111
